# Joint analysis of multiple phenotypes for extremely unbalanced case–control association studies using multi-layer network

**DOI:** 10.1093/bioinformatics/btad707

**Published:** 2023-11-22

**Authors:** Hongjing Xie, Xuewei Cao, Shuanglin Zhang, Qiuying Sha

**Affiliations:** Department of Mathematical Sciences, Michigan Technological University, Houghton, MI 49931, United States; Department of Mathematical Sciences, Michigan Technological University, Houghton, MI 49931, United States; Department of Mathematical Sciences, Michigan Technological University, Houghton, MI 49931, United States; Department of Mathematical Sciences, Michigan Technological University, Houghton, MI 49931, United States

## Abstract

**Motivation:**

Genome-wide association studies is an essential tool for analyzing associations between phenotypes and single nucleotide polymorphisms (SNPs). Most of binary phenotypes in large biobanks are extremely unbalanced, which leads to inflated type I error rates for many widely used association tests for joint analysis of multiple phenotypes. In this article, we first propose a novel method to construct a Multi-Layer Network (MLN) using individuals with at least one case status among all phenotypes. Then, we introduce a computationally efficient community detection method to group phenotypes into disjoint clusters based on the MLN. Finally, we propose a novel approach, MLN with Omnibus (MLN-O), to jointly analyse the association between phenotypes and a SNP. MLN-O uses the score test to test the association of each merged phenotype in a cluster and a SNP, then uses the Omnibus test to obtain an overall test statistic to test the association between all phenotypes and a SNP.

**Results:**

We conduct extensive simulation studies to reveal that the proposed approach can control type I error rates and is more powerful than some existing methods. Meanwhile, we apply the proposed method to a real data set in the UK Biobank. Using phenotypes in Chapter XIII (Diseases of the musculoskeletal system and connective tissue) in the UK Biobank, we find that MLN-O identifies more significant SNPs than other methods we compare with.

**Availability and implementation:**

https://github.com/Hongjing-Xie/Multi-Layer-Network-with-Omnibus-MLN-O.

## 1 Introduction

A genome-wide association studies (GWAS) is defined by the National Institutes of Health as an approach that involves rapidly scanning markers across the complete sets of DNA, or genomes, of many people to find genetic variations associated with a particular disease (National Institutes of Health 2007). Common statistical methods usually test the association between a single phenotype and multiple single nucleotide polymorphisms (SNPs), which means, only one phenotype is tested at a time. The joint analysis between multiple phenotypes and a SNP can increase the power of detecting significant SNPs and enhance computing efficiency in comparison with the analysis between a single phenotype and a SNP (Ferreira and Purcell 2009, Im *et al.* 2012, O’Reilly *et al.* 2012, Aschard *et al.* 2014, Kim *et al.* 2015, Zhu *et al.* 2015, Sha *et al.* 2019). Recently, more and more joint analyses of multiple phenotypes have been put forward to analyse the relationship between multiple related phenotypes and a SNP.

Previous studies revealed that some SNPs are significantly associated with multiple phenotypes (Lobo 2008, Kent Jr 2009, Stearns 2010, Sivakumaran *et al.* 2011, Solovieff *et al.* 2013, Liang *et al.* 2018, Geiler-Samerotte *et al.* 2020, Liu *et al.* 2021) and joint analysis of multiple phenotypes is in accordance with biology (Van der Sluis *et al.* 2013), therefore, many statistical methods for joint analyses of multiple phenotypes have been proposed (Galesloot *et al.* 2014). Briefly, these methods can be mainly divided into three categories. The first category is based on regression methods such as generalized linear mixed effects model (Fitzmaurice and Laird 1993), generalized estimating equations (Liang and Zeger 1986), Multiphen (O’Reilly *et al.* 2012), and multivariate analysis of variance (MANOVA) (Cole *et al.* 1994) which is suitable for categorical independent variables. However, MANOVA might lose power when a SNP is associated with all phenotypes. Debashree Ray *et al.* proposed a unified score-based test statistic, USAT, that performs better than MANOVA in such situations and nearly as well as MANOVA elsewhere (Ray *et al.* 2016).

The second category is combining test statistics from univariate analyses, which is simpler and more flexible than other methods especially when the dependent variables include continuous, discrete, and survival data (Yang and Wang 2012). For example, Sophie van der Sluis (Van der Sluis *et al.* 2013) developed a method which is called the Trait-based Association Test that uses the Extended Simes procedure (TATES). The TATES method combines *P*-values obtained from standard univariate GWAS to acquire one trait-based *P*-value while correcting for correlations between components (Van der Sluis *et al.* 2013). Zhang et al. (2014) presented a category of the professed sum of powered score (SPU) tests by incorporating all score test statistics from univariate tests. Xu et al. (2003) developed the Wald Chi-squared type statistic by taking a quadratic form of the vector of the univariate association test statistic.

The third category is based on variable reduction methods which are, in general, only applicable to multiple continuous phenotypes that are approximately normally distributed (Yang and Wang 2012). For instance, the popular dimension reduction approach, principle component analysis of phenotypes (Aschard *et al.* 2014), and canonical correlation analysis (Tang and Ferreira 2012). Due to the relatedness of the multiple phenotypes, dimension reduction of the dependent variables becomes more essential. There are more dimension reduction methods emerged recently, such as the clustering linear combination (CLC) method (Sha *et al.* 2019), computationally efficient CLC (ceCLC) (Wang *et al.* 2022), hierarchical clustering method (HCM) (Liang *et al.* 2018), and an agglomerative nesting clustering algorithm for phenotypic dimension reduction in the joint analysis of multiple phenotypes (AGNEP) (Liu *et al.* 2021). But many of these methods are time consuming, and CLC, ceCLC, HCM, and AGNEP are not suitable for extremely unbalanced case–control studies (Cao *et al.* 2023).

In this article, we propose a novel dimension reduction method called MLN with Omnibus (MLN-O) by utilizing Multi-Layer Network (MLN) and Omnibus test statistics to test the association between multiple phenotypes and a SNP. MLN is designed for dimension reduction of correlated and extremely unbalanced case–control phenotypes. To build MLN, we only consider individuals with at least one case status for all phenotypes which can significantly reduce the running time of MLN construction and community detection when the case-control ratios of phenotypes are extremely unbalanced.

## 2 Materials and methods

Suppose there are n unrelated individuals with K correlated phenotypes and a SNP. The *k*th (k=1,2,…,K) binary phenotype of the *i*th individual is denoted by $y_{ik}$, where $y_{ik}=0$ or 1 represents the control or case status of the individual respectively. The SNP of the *i*th individual is represented by $x_{i}$, which can take one of the three values, 0, 1, and 2, to indicate the number of minor alleles at the SNP. Our proposed approach, MLN-O, involves the following three steps.

### 2.1 Step 1. Construct an MLN based on phenotypes

In the first step, we introduce a novel method to construct an MLN using individuals with at least one case status among all phenotypes. For each layer (individual) i, the (*j*,*k*)th element of the adjacency matrix $A^{i}$ is given by


(1) $$A_{jk}^{i}=\{\begin{array}{c} 1,\;y_{ij}\times y_{ik}=1\\ 0,\;\text{otherwise} \end{array},$$

with i=1,…,n;j,k=1,…,K. The element $A_{jk}^{i}=1$ indicates that individual i has a case status for both *j*th and *k*th phenotypes. Then, we combine $A^{i}$ for all n individuals to obtain the overall adjacency matrix A with multiple layers, i.e. $A=\sum_{i=1}^{n}A^{i}$, where $A_{jk}=a$ means that there are a individuals with a case status for both *j*th and *k*th phenotypes. We then define the transformed similarity matrix W as $W=\text{diag}{\left(A\right)}^{-1/2}\cdot A\cdot \;\text{diag}{\left(A\right)}^{-1/2}$.

Note that the construction of MLN using all individuals is equivalent to constructing it solely using individuals who have at least one case status among all phenotypes. If the *i*th individual with a control status for all phenotypes, $y_{ik}=0$ for k=1,2,…,K, and $A_{jk}^{i}=0$ for all pairs of (j,k). So, the *i*th individual does not contribute to the construction of the MLN. If we construct the MLN excluding the individuals with all control status, the computational time can be significantly reduced.

The advantage of the MLN is obvious for analyzing the extremely unbalanced case–control phenotypes. The construction of MLN is intuitive. Each individual has a single layer network, we combine all of the networks of the individuals to get the MLN. It uses the number of edges between different phenotypes to represent the intensity of the connection among all phenotypes instead of the correlation of the phenotypes of all individuals in a dataset. MLN enhances the connectivity of phenotypes. It only considers individuals with at least one case status but does not consider individuals without any diseases. Because they do not carry any information to reveal the clustering structures among phenotypes.

### 2.2 Step 2. Cluster phenotypes by a community detection method based on the transformed similarity matrix

Based on the transformed similarity matrix W, we propose the following community detection method based on modularity measurement (Fortunato 2010, Malliaros and Vazirgiannis 2013). We divide the K phenotypes into $k_{0}$ clusters $\left(k_{0}=1,2,\ldots ,K\right)$ using a complete hierarchical clustering method with similarity matrix W and build a K×K connectivity matrix $C^{\left(k_{0}\right)}$, where the (*j*,*k*)th element is given by


$$C_{j,k}^{\left(k_{0}\right)}=\left\{ \begin{array}{c} 1,\;\mathrm{if}\;j\;\mathrm{and}\;k\;\mathrm{are}\;\mathrm{in}\;\mathrm{the}\;\mathrm{same}\;\mathrm{community}\\ 0,\;\text{otherwise} \end{array}\right..$$

Then we calculate the modularity of the network with $k_{0}$ clusters, denoted as $Q_{k_{0}}$, and it is given by


(2) $$Q_{k_{0}}=\frac{1}{2D}\sum_{j,k=1}(W_{j,k}-\frac{d_{j}d_{k}}{2D})\;C_{j,k}^{\left(k_{0}\right)},$$

where $d_{j}=\sum_{k}W_{j,k}$ represents the total degrees of node j; and $D=\sum_{j}d_{j}/2$ stands for the total number of edges in the MLN.

Modularity is a measure of the structure of a network, which measures how well the network is divided into different modules or clusters. A network with high modularity has dense connections between the phenotypes within clusters but sparse connections between phenotypes in different clusters. Using the modularity metric to decide the optimal number of clusters is straightforward and computationally efficient. We determine the optimal number of clusters as $L=\arg\max_{l=1,\ldots ,K}\{Q_{l}\}$.

After we cluster the total K phenotypes into L clusters, where 1≤L≤K, the phenotypes in the same cluster are merged into a single dichotomous phenotype. Suppose the *l*th cluster has $K_{l}$ phenotypes, $l_{1}^{th},\ldots ,\;\text{and}\;l_{K_{l}}^{th}$ phenotypes, then for the *i*th individual, the merged phenotype is defined as $Y_{il}=\max(y_{il_{1}},\;y_{il_{2}},\ldots \;,y_{il_{K_{l}}})$ for i=1,…,n and l=1,…,L.

### 2.3 Step 3. Test the association between phenotypes and a SNP

We first assume that there are no covariates. To test the association between phenotypes in each cluster and a SNP, we consider the merged phenotype in a cluster and a SNP following the generalized linear model (Wu *et al.* 2011, Pan *et al.* 2014)


(3) g(E[Yil|x]i)=β0l+β1lxi for i=1,2,…,n; l=1,2,…,L

where g(⋅) is a monotonic link function (Li *et al.* 2020). The link function takes the identity function under the linear regression model framework for quantitative or continuous phenotypes and takes the logit function under the logistic regression model framework for qualitative or binary phenotypes.

If there are p covariates $z_{i1},z_{i1},\ldots ,z_{ip}$ being considered for the *i*th individual, we can adjust all phenotypes and the genotype by the covariates via the following linear regression models (Price *et al.* 2010),


$$x_{i}=\alpha_{0}+\alpha_{1}z_{i1}+\cdots +\alpha_{p}z_{ip}+\varepsilon_{i},$$

$$Y_{il}=\gamma_{0l}+\gamma_{1l}z_{i1}+\gamma_{2l}z_{i2}+\cdots +\gamma_{pl}z_{ip}+\tau_{il}.$$

That means we use the residuals of the above linear models instead of the original phenotypes and genotype to perform downstream analysis. It’s worth noting that after we adjust the phenotypes for the covariates, $Y_{il}$ becomes a continuous phenotype.

We propose the following Omnibus statistic based on the MLN (MLN-O) to test the association between phenotypes and a SNP. To test the associations between the *l*th merged phenotype and a SNP under model (3), we use the score test statistic given by


$$T_{l}=\frac{\sum_{i=1}^{n}\left(x_{i}-\bar{x})(Y_{il}-{\bar{Y}}_{l}\right)}{\sqrt{n{\sum_{i=1}^{n}\left(x_{i}-\bar{x}\right)}^{2}\;\sum_{i=1}^{n}{\left(Y_{il}-{\bar{Y}}_{l}\right)}^{2}}},$$

where $\bar{x}=\sum_{i=1}^{n}x_{i}/n$ and ${\bar{Y}}_{l}=\sum_{i=1}^{n}Y_{il}/n$ for l=1,…,L. To test the association between phenotypes in all clusters and a SNP, we use the Omnibus statistic to combine the score test statistics $T_{1},\ldots ,T_{L}$(Sha *et al.* 2019). The Omnibus statistic is given by


(4) $$T_{\mathrm{MLN}-O}=(T_{1},\;T_{2},\;\ldots \;,\;T_{L})\Sigma^{-1}{\left(T_{1},\;T_{2},\ldots ,\;T_{L}\right)}^{T},$$

where $\Sigma =\left(\Sigma_{kl}\right)$ is the correlation matrix of $\left(T_{1},\;T_{2},\;\ldots \;,\;T_{L}\right)$. Based on Sha *et al.* (2019), we can use the correlation matrix of the L merged phenotypes to estimate Σ, where the (*k*,*l*)th element of Σ is estimated by ${\hat{\Sigma }}_{kl}=\sum_{i=1}^{n}\left(Y_{ik}-{\bar{Y}}_{k}\right)\left(Y_{il}-{\bar{Y}}_{l}\right)/\sqrt{\sum_{i=1}^{n}{\left(Y_{ik}-{\bar{Y}}_{k}\right)}^{2}\sum_{i=1}^{n}{\left(Y_{il}-{\bar{Y}}_{l}\right)}^{2}}$. Then, $T_{\mathrm{MLN}-O}$ follows a Chi-square distribution with L degrees of freedom.

## 3 Comparison methods

We compare MLN-O with the following four commonly used methods for multiple phenotypes association studies.

**MANOVA** (Cole *et al.* 1994): MANOVA estimates the association between categorical independent variables and multiple response variables simultaneously. The test statistic (Wilk’s Lambda) of MANOVA, $T_{\mathrm{MANOVA}}$, measures the proportion of variance explained by the dependent variable which is equivalent to the likelihood ratio test that can be approximated with an asymptotic $\chi^{2}$ distribution with K degree of freedom, where K is the number of phenotypes.

**USAT** (Ray *et al.* 2016): UAST is the weighted summation of the Sum of Squared Score (SSU) test statistic (Yang and Wang 2012) and MANOVA test statistic. Suppose $T_{\mathrm{SSU}}$ represents the test statistic of SSU, which follows a shifted scaled $\chi^{2}$ distribution with 1 degree of freedom. USAT is defined as $T_{\mathrm{USAT}}=\underset{0\leq w\leq 1}{\min}p_{w}$, where $p_{w}$ is the *P*-value of the test statistic $T_{w}=wT_{\mathrm{MANOVA}}+(1-w)T_{\mathrm{SSU}}$ and w is a tuning parameter.

**TATES** (Van der Sluis *et al.* 2013): For each phenotype among multiple correlated phenotypes, TATES adjusts for correlations between phenotypes, then integrates *P*-values estimated from traditional univariate GWAS for a SNP and each of phenotypes. The integrated phenotype-based *P*-value $P_{\mathrm{TATES}}$ is obtained from the extended Simes procedure with $p_{\mathrm{TATES}}=\min\left(K_{e}p_{\left(i\right)}/K_{ei}\right)$, where $K_{e}$ stands for the effective number of all K*P*-values for a given SNP, and $K_{ei}$ is the effective number of *P*-values of the top  (i=1,2,…,K)*P*-values, and $p_{\left(i\right)}$ is the $i^{th}$ ordered *P*-value.

**MultiPhen** (O’Reilly *et al.* 2012): MultiPhen applies the proportional odds logistic regression to regress genotype of a SNP on multiple phenotypes to test whether effect sizes of all phenotypes are significantly different from zero. The resulting test statistic is based on the likelihood ratio test that asymptotically follows a $\chi^{2}$ distribution with K degrees of freedom, where K is the number of phenotypes.

## 4 Simulation studies

To evaluate the type I error rate and power of MLN-O, we generate genotypes according to the minor allele frequency (MAF) and assume Hardy Weinberg equilibrium. To generate a dichotomous disease affection status, we first generate quantitative phenotypes similar to that in Wang *et al.* (Wang *et al.* 2018). Then, we use a liability threshold model based on these quantitative phenotypes to define disease affection status. That is, the top nr quantitative phenotypes are defined to be affected in the ordered phenotypes in decreasing order, where n is the number of samples and r is the case–control ratio. To generate the binary phenotypes with the extremely unbalanced case–control ratios, we use r=0.001 and 0.002. In the following, we describe how to generate quantitative phenotypes by the factor model


$$y=\lambda x+c\gamma f+\sqrt{1-c^{2}}\varepsilon ,$$

where $y=(y_{1},y_{2},\ldots ,y_{K})$ is a vector of phenotypes, x is the genotype at a SNP of interest; $\lambda ={\left(\lambda_{1},\lambda_{2},\ldots ,\lambda_{K}\right)}^{T}$ is the vector of effect sizes of the SNP on the phenotypes; $f=(f_{1},f_{2},\ldots ,f_{R})$ is a vector of factors with R elements and f:MVN(0,Σ), where Σ=(1−ρ)I+ρJ, J is a matrix with elements of 1 in size R×R, I is the identity matrix in the same size with J, and ρ is the correlation between factors; γ is a K by R matrix; c is a constant number; and $\varepsilon ={\left(\varepsilon_{1},\varepsilon_{2},\ldots ,\varepsilon_{K}\right)}^{T}$ is a vector of residuals, $\varepsilon_{1},\varepsilon_{2},\ldots ,\varepsilon_{K}$ are independent, and $\varepsilon_{k}:N(0,1)$ for k=1,2,…,K.

Based on the factor model, we consider the following four models in which the within-factor correlation is $c^{2}$ and the between-factor correlation is $\rho c^{2}$. The phenotypic correlation configuration mimic that of UK10K (UK10K Consortium 2015), by way of explanation, the phenotypes are split into several phenotype blocks (phenotype factors) and the within-factor correlation is larger than the between-factor correlation (Sha *et al.* 2019).

**Model 1**: There are five factors (R=5) and genotypes impact on two factors with the same effect size. That is, $\lambda =(\beta_{11},\ldots ,\beta_{1k},\ldots ,\beta_{41},\ldots ,\beta_{4k},\beta_{51},\ldots ,\beta_{5k})$, where k=K/5, and $\gamma =\mathrm{Bdiag}(1_{K/5},\ldots ,1_{K/5})$ is a block diagonal matrix, and $\beta_{11}=\cdots =\beta_{1k}=\beta_{21}=\cdots =\beta_{2k}=\beta_{31}=\cdots =\beta_{3k}=0$, $\beta_{41}=\cdots =\beta_{4k}=\beta_{51}=\cdots =\beta_{5k}=\beta .$

**Model 2**: There are five factors (R=5) and genotypes impact on two factors with the same effect size, but opposite directions. That is, $\gamma =\mathrm{Bdiag}(1_{K/5},\ldots ,1_{K/5})$ is a block diagonal matrix, and $\lambda =(\beta_{11},\ldots ,\beta_{1k},\ldots ,\beta_{41},\ldots ,\beta_{4k},\beta_{51},\ldots ,\beta_{5k})$, where k=K/5, $\beta_{11}=\cdots =\beta_{1k}=\beta_{21}=\cdots =\beta_{2k}=\beta_{31}=\cdots =\beta_{3k}=0$, $\beta_{41}=\cdots =\beta_{4k}=-\beta$, and $\beta_{51}=\cdots =\beta_{5k}=\beta .$

**Model 3**: There are five factors (R=5) and genotypes impact on two factors with different effect sizes and opposite directions. That is, $\gamma =\mathrm{Bdiag}(1_{K/5},\ldots ,1_{K/5})$ is a block diagonal matrix, and $\lambda =(\beta_{11},\ldots ,\beta_{1k},\ldots .,\beta_{41},\ldots ,\beta_{4k},\beta_{51},\ldots ,\beta_{5k})$, where k=K/5, $\beta_{11}=\cdots =\beta_{1k}=\beta_{21}=\cdots =\beta_{2k}=\beta_{31}=\cdots =\beta_{3k}=0$, $\beta_{41}=\cdots =\beta_{4k}=-\beta$, and $\beta_{51}=\cdots =\beta_{5k}=2\beta /\left(k+1\right)(1,2,\ldots ,k).$

**Model 4**: There are ten factors (R=10) and genotypes impact on four factors. That is, $\gamma =\mathrm{Bdiag}(1_{K/10},\ldots ,1_{K/10})$, k=K/10, $\lambda =(\beta_{11},\ldots ,\beta_{1k},\beta_{21},\ldots ,\beta_{2k},\ldots ,\beta_{91},\ldots ,\beta_{9k},\beta_{10,1},\ldots ,\beta_{10,k})$, where $\beta_{11}=\cdots =\beta_{1k}=\beta_{21}=\cdots =\beta_{2k}=\cdots =\beta_{61}=\cdots =\beta_{6k}=0$, $\beta_{71}=\cdots =\beta_{7k}=-\beta /\left(k/2+1\right)(1,2,\ldots ,k/2,k/2,\ldots ,2,1)$, $\beta_{81}=\cdots =\beta_{8k}=\beta /\left(k/2+1\right)(1,2,\ldots ,k/2,k/2,\ldots ,2,1)$, $\beta_{91}=\cdots =\beta_{9k}=-\beta$, and $\beta_{10,1}=\cdots =\beta_{10,k}=\beta .$

In the simulation studies, we set MAF=0.3, the between-factor correlation $\rho c^{2}=0.24$, and the within-factor correlation $c^{2}=0.4$. To evaluate the type I error rates of the method we proposed, we let β=0. To evaluate the power, we let β≠0. To generate phenotypes with extremely unbalanced case–control ratios, we use two different case–control ratios, 0.001 and 0.002.

## 5 Results

To evaluate the type I error rate, we set β=0 and the number of phenotypes K=100. Table 1 displays type I error rates of the five methods, MLN-O, USAT, MultiPhen, MAMOVA, and TATES, under model 1 with two case–control ratios (0.001 and 0.002), two sample sizes (20 000 and 30 000), and four significance levels (0.05, 0.01, 0.001 and 0.0001). For significance levels of 0.05, 0.01, 0.001 and 0.0001, the 95% confident intervals (CIs) are (0.04865, 0.05135), (0.00938, 0.01062), (0.0008, 0.0012), (0.00004, 0.00016) under 100 000 replicates, respectively. The values in boldface indicate that the type I error rates are out of control. From Table 1, we can observe that our proposed method MLN-O, USAT, and MANOVA can control type I error rates and USAT is conservative in all scenarios. MultiPhen cannot control type I error rates in all scenarios except at significance level α=0.0001. Meanwhile, TATES cannot control type I error rates in some scenarios. We observe the similar results under models 2–4 in Supplementary Tables S1–S3.

**Table 1.** The estimated type I error rates for two extremely case–control ratios (ratio = 0.001 and ratio = 0.002), two different sample sizes (*n* = 20 000 and *n* = 30 000) under model 1 at different significant levels with 100 000 replicates.^a^

Ratio	Sample	α-level	MLN-O	USAT	MANOVA	MultiPhen	TATES
0.001	20 000	0.05	0.04979	0.03131	0.04911	**0.07791**	0.04361
		0.01	0.00959	0.00594	0.00946	**0.01770**	0.00887
		0.001	0.00099	0.00057	0.00079	**0.00199**	**0.00227**
		0.0001	7.00E−05	3.00E−05	6.00E−05	0.00022	0.00015
	30 000	0.05	0.05115	0.03114	0.04991	**0.06089**	0.04901
		0.01	0.01009	0.00622	0.00962	**0.01252**	0.01049
		0.001	0.00099	6.00E−04	0.00088	0.00128	0.00127
		0.0001	0.00011	4.00E−05	7.00E−05	8.00E−05	0.00012
0.002	20 000	0.05	0.04964	0.03191	0.04997	**0.0659**	0.04435
		0.01	0.01001	0.00648	0.0096	**0.01452**	**0.01169**
		0.001	0.00110	0.00066	0.00086	**0.00135**	0.00124
		0.0001	8.00E−05	0.00014	0.00013	0.00016	0.00016
	30 000	0.05	0.04952	0.03082	0.04909	**0.06780**	0.04376
		0.01	0.00999	0.00627	0.00934	**0.01475**	**0.01372**
		0.001	0.00092	0.00059	0.00095	**0.00185**	**0.00172**
		0.0001	0.00011	7.00E−05	7.00E−05	0.00014	1.00E−04

a The bold-faced values indicate the *P*-values beyond the upper bound of the corresponding 95% CIs.

**Figure 1.**
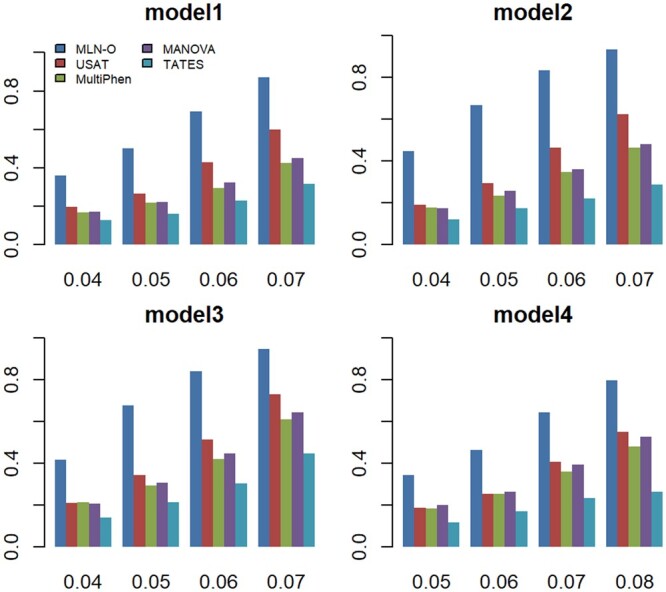
Power comparisons of the five tests (MLN-O, USAT, MANOVA, MultiPhen, and TATES) for 100 binary phenotypes. The sample size is 20 000 and the case–control ratio is 0.002. The between-factor correlation is 0.24 and the within-factor correlation is 0.4.

We compare the power of MLN-O with the four comparing methods, USAT, MultiPhen, MAMOVA, and TATES. We consider the same settings for power comparison as that for type I error evaluations, which contain two case–control ratios (0.001 and 0.002) along with two sample sizes (20 000 and 30 000) for β≠0. Figure 1 shows the power comparisons of the five tests (MLN-O, USAT, MultiPhen, MAMOVA, TATES) for 100 binary phenotypes. The sample size is 20 000 and the case–control ratio is 0.002. We can observe that the method we proposed, MLN-O, has the highest power and TATES has the lowest power in all settings under all models. The other three methods, USAT, MultiPhen and MANOVA, have similar powers, but USAT outperforms MultiPhen and MANOVA in most of the settings; MANOVA has slightly higher power compared with MultiPhen. Supplementary Fig. S1 shows the power comparisons of all methods for 100 binary phenotypes with the sample size 20 000 and the case–control ratio 0.001. Supplementary Figs S2 and S3 display the power comparison results for the sample size 30 000 under the case–control ratio 0.001 and 0.002, respectively. The patterns of the powers shown in Supplementary Figs S1–S3 are similar to what we observe in Fig. 1.

## 6 Applications to UK Biobank

The UK Biobank cohort contains about 500 000 participants in the United Kingdom (UK) (Bycroft *et al.* 2018) and the genome-wide genotyping was performed using the UK Biobank Axiom Array. Approximately 850 000 variants were directly measured, with more than 90 million variants imputed using the Haplotype Reference Consortium and UK10K and 1000 Genomes reference panels (https://www.ukbiobank.ac.uk/enable-your-research/about-our-data/genetic-data). The UK Biobank has already inspired many researchers to explore the associations between human genetic variation and disease, and their connection with a wide range of environmental and lifestyle factors (Bycroft *et al.* 2018). There are more than almost 3000 papers published using the UK Biobank data (https://www.ukbiobank.ac.uk/enable-your-research/publications) so far. Various studies using UK Biobank data have successfully identified thousands of genetic variants, such as SNPs, that are associated with human traits and diseases (Solovieff *et al.* 2013).

The principal limitation of UK Biobank data is that most of the binary phenotypes are extremely unbalanced and many of the current methods for association studies are not suitable for this kind of phenotypes. Our proposed MLN-O method is applicable to the extremely unbalanced binary phenotypes. Following the phenotype preprocess introduced by Liang et al. (2022), we consider 72 level 3 phenotypes with the number of cases >200 in Chapter XIII (Diseases of the musculoskeletal system and connective tissue) (Field ID = 41202) in UK Biobank. The phenotypes’ codes are based on the International Classification of Diseases, the 10th Revision (ICD-10) codes. We also use the quality controls (QCs) for both SNPs and individuals using PLINK 1.9 (https://www.cog-genomics.org/plink/1.9/) to select 288 647 SNPs that is described in Liang et al. (2022). After preprocessing phenotype data and performing QCs, 322 607 individuals are considered together with 72 phenotypes, and 13 covariates containing the first 10 principal components for adjusting the population stratification, genotype array, sex, and age. These phenotypes are extremely unbalanced. The minimum case–control ratio among these 72 phenotypes is 0.0006354481 (M25.4) and the maximum case–control ratio is 0.0378727058 (M17.9). We first construct the MLN of these 72 phenotypes. After applying the proposed community detection method to the MLN, these 72 phenotypes can be partitioned into 19 disjoint clusters (Supplementary Table S4).

**Figure 2.**
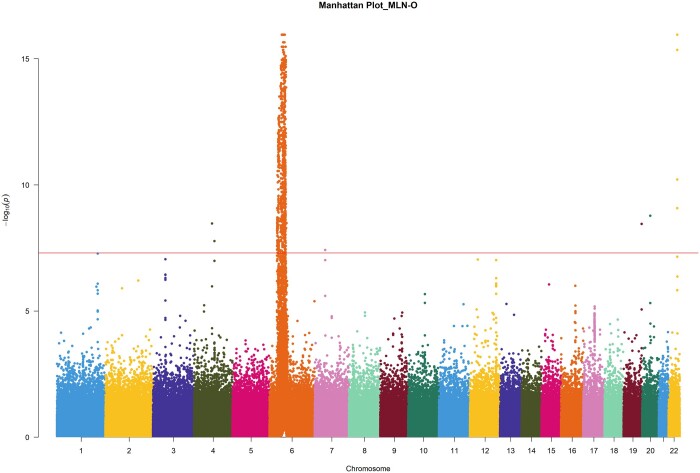
Manhattan plot of the negative log-transformed *P*-values in the real data analysis by MLN-O against base pair positions for 22 autosomes. The horizontal line represents the GWAS significance level.

We apply the five methods to the UK Biobank data set described above. Figure 2 is the Manhattan plot of MLN-O which displays most of the significant SNPs on chromosome 6. In the Manhattan plot, the vertical axis is the negative logarithm of the association *P*-value for each SNP and the horizontal axis represents the genomic coordinates including the 22 autosomes. The extent of each chromosome is shown by each block with different colors. At the significance level $\alpha =5\times 10^{-8}$, the total number of SNPs identified by MLN-O is 1030, which are the dots above the horizontal line in Fig. 2. To compare our method with other four methods in the read data analysis, we also apply the other four methods, USAT, TATES, MANOVA, and MultiPhen, on the same UK Biobank data set. MANOVA detects 648 significant SNPs, followed by USAT (627). The other two methods, MultiPhen and TATES, identify 610 and 619 significant SNPs, respectively. The Manhattan plots of these four methods are shown in Supplementary Fig. S4. We can see from Supplementary Fig. S4, the majority of the significant SNPs identified by each method are on chromosome 6.

**Figure 3.**
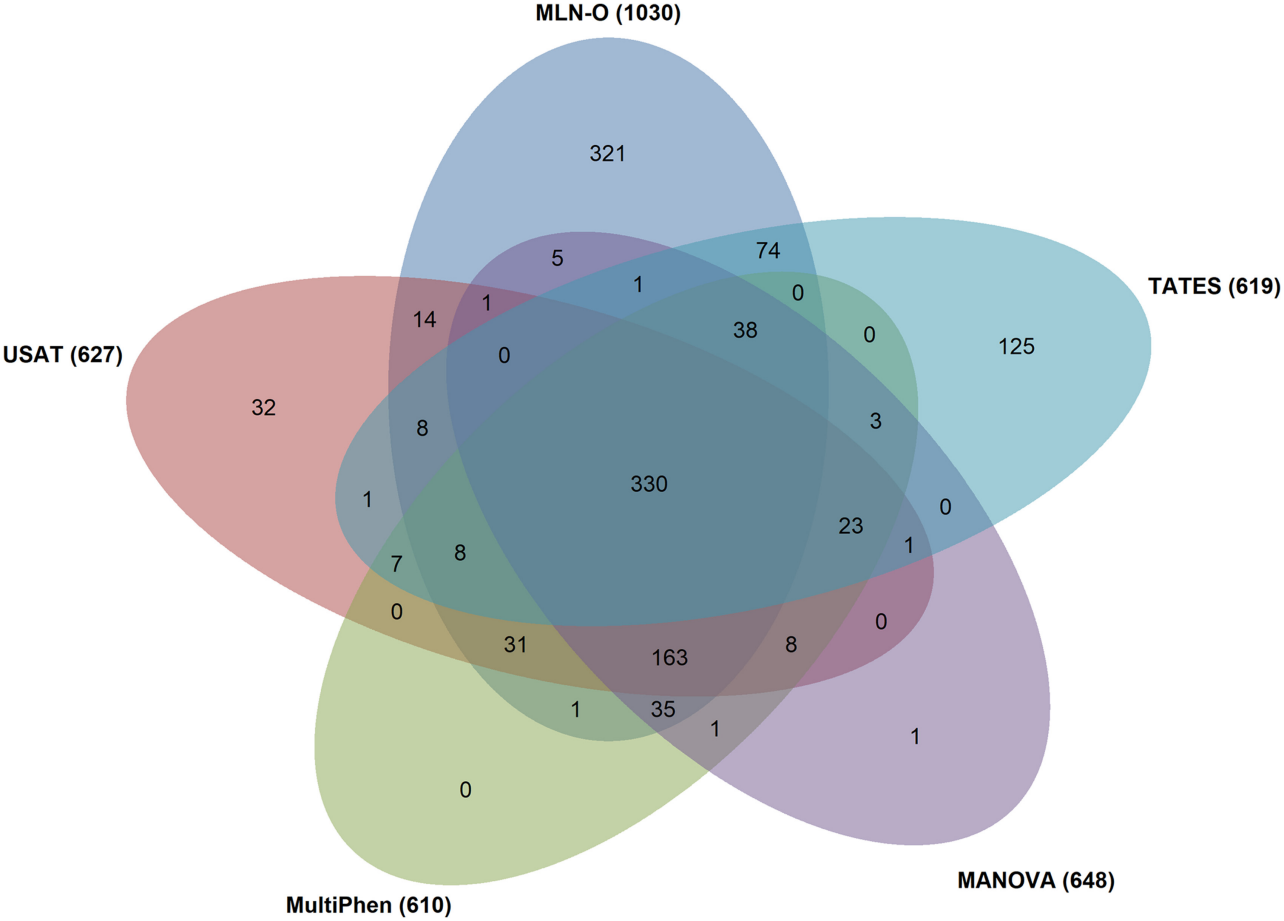
Venn diagram of significant SNPs identified by the five methods for Category XIII (diseases of the musculoskeletal system and connective tissue) in UK Biobank.

To visualize the intersecting sets of significant SNPs identified by the five methods, we implement the Venn diagram in Fig. 3. From the Venn diagram, we can see that there are 330 significant SNPs found by all five methods and 321 SNPs only identified by MLN-O. Among 321 SNPs detected by MLN-O, two SNPs are reported in the GWAS Catalog that are significantly associated with the phenotypes in Chapter XIII (Diseases of the musculoskeletal system and connective tissue): rs3130340 is associated with Bone mineral density (spine) (M85.8) reported by Styrkarsdottir et al. (2008) and rs3130320 is associated with Systemic lupus erythematosus (M32.9) reported by Chung *et al.* (2011). We also map 321 SNPs to gene regions (±0 kb window) and there are 248 SNPs mapped to 119 genes. Among these 119 genes, 50 genes are associated with the phenotypes in Chapter XIII reported in the GWAS Catalog. Supplementary Table S5 shows the *P*-values of the five methods for the SNPs identified by MLN-O, the mapped genes, and references for the mapped genes that are associated with the diseases in Chapter XIII reported in the GWAS Catalog.

## 7. Discussions

To date, statistical methods for joint analysis of multiple phenotypes becomes an essential tool to increase statistical power for detecting significant associations between multiple phenotypes and SNPs. In this paper, we propose a method, MLN-O, for testing the association between a SNP and multiple correlated binary phenotypes with extremely unbalanced case–control ratios. Through considerable simulation studies, we show that the type I error rates can be correctly controlled by MLN-O. As for the power comparison in simulation studies, MLN-O has the highest power among all the methods we compared in all simulation settings under the four models. In the real data analysis, MLN-O identifies much more significant SNPs associated with the 72 correlated diseases of the musculoskeletal system and connective tissue compared with the other four methods. MLN-O detects 321 unique significant SNPs and half of them are reported in the GWAS Catalog to be associated with the corresponding diseases. We also conclude that a large amount of the significant SNPs associated with the 72 correlated diseases of the musculoskeletal system and connective tissue are enriched on chromosome 6.

There are some advantages of MLN-O. First, the construction of MLN is based on an individual’s case–control status to reveal the relationships among all phenotypes we considered. MLN uses the number of edges between different phenotypes to represent the intensity of the connection among all phenotypes instead of the correlation of the phenotypes of all individuals in a dataset. Second, community detection based on MLN is computationally efficient. The phenotype clustering using MLN is more robust and time efficient because of the dimension reduction. Third, clustering based on the MLN is suitable for extremely unbalanced case–control phenotypes no matter how small the case–control ratio is as long as the sample size is large enough since the consideration only focuses on the individuals with case-statues when clustering the correlated phenotypes. In fact, the smaller the case–control ratio the faster the clustering. Fourth, after clustering and merging the correlated phenotypes, the case–control ratios of the merged phenotypes are increased. Data analysis using MLN-O to the UK Biobank shows that MLN-O identifies much more significant SNPs than other comparison methods. From simulation studies and real data analysis, we can see that clustering phenotypes based on network is a competitive method. In the future, we can consider integrating genetic information to cluster phenotypes.

If the number of clusters in MLN is relatively large, the omnibus test have a large number of degrees of freedom, which may impact the validity of the test. To assess the validity of MLN-O in a scenario involving a large number of clusters, we simulate an additional model (Model 5) with K=100 binary phenotypes in 50 groups. There are 50 factors and genotypes impact on ten factors with the same effect size, but opposite directions. That is, $\lambda =(\beta_{11},\beta_{12},\beta_{21},\beta_{22},\ldots ,\beta_{50\;1},\beta_{50\;2})$ and $\gamma =\mathrm{Bdiag}(1_{K/50},\ldots ,1_{K/50})$ is a block diagonal matrix, where $\beta_{11}=\beta_{12}=\cdots =\beta_{40,1}=\beta_{40,2}=0$; $\beta_{41,1}=\beta_{41,2}=\cdots =\beta_{45,1}=\beta_{45,2}=\beta$; $\beta_{46,1}=\beta_{46,2}=\cdots =\beta_{50,1}=\beta_{50,2}=-\beta$. Supplementary Table S6 shows the estimated type I error rates of the five methods under Model 5 at different significant levels with 100 000 replicates. Similar to models 1–4, our proposed method MLN-O, USAT, and MANOVA can control type I error rates, but MultiPhen cannot control type I error rates in all scenarios and TATES cannot control type I error rates in some scenarios. The results show that the omnibus test is also a valid test with a large number of degrees of freedom.

In our proposed MLN-O, we use omnibus statistic to test the association between the merged phenotypes with a SNP in step 3. Actually, any multiple phenotype association test can be used to substitute the omnibus statistic, such as MANOVA and USAT. It is also important to emphasize that the proposed method, MLN-O, is specifically designed to address extremely unbalanced case–control association studies. In scenarios where binary phenotypes are balanced, the majority of the merged phenotypes would hold a value of 1. So, the proposed method is not suitable for such cases. Therefore, we recommend applying our proposed MLN-O method exclusively to only the situation involving multiple binary phenotypes with extremely unbalanced case–control.

## Supplementary Material

btad707_Supplementary_Data

## Data Availability

The data underlying this article were provided by the UK Biobank under applications. Data will be shared on request to the corresponding author with permission of the UK Biobank.
