# Supplementary material for: Joint analysis of multiple phenotypes for extremely unbalanced case–control association studies using multi-layer network
Source: Bioinformatics. 2023 Nov 22;39(12):btad707. doi: 10.1093/bioinformatics/btad707 (PMC10697735; doi:10.1093/bioinformatics/btad707)
Supplement: btad707_Supplementary_Data [file btad707_supplementary_data.pdf]

Supplementary Information

Joint analysis of multiple phenotypes for extremely unbalanced case-control association studies using multi-layer network

Hongjing Xie[1], Xuewei Cao[1], Shuanglin Zhang[1], Qiuying Sha[1,*]

[1]Department of Mathematical Sciences, Michigan Technological University, Houghton, Michigan, USA

*To whom correspondence should be addressed.

Table S1. The estimated type I error rates for two extremely case-control ratios (0.001 and 0.002) with two different sample sizes (20,000 and 30,000) under model 2 at different significant levels with 100,000 replicates.

	Model 2		MLN-O	USAT	MANOVA	MultiPhen	TATES
C-C Ratio	Sample	α-level					
0.001	20,000	0.05	0.04940	0.03061	0.04795	**0.07809**	0.04293
		0.01	0.00992	0.00623	0.00920	**0.01819**	0.00870
		0.001	0.00099	0.00062	8.00E-04	**0.00220**	**0.00201**
		0.0001	0.00012	8.00E-05	1.00E-04	**0.00027**	0.00017
	30,000	0.05	0.04915	0.03222	0.05004	**0.06191**	0.04845
		0.01	0.01024	0.00709	0.01032	**0.01366**	0.01044
		0.001	0.00098	0.00084	0.00096	**0.00151**	0.00121
		0.0001	8.00E-05	7.00E-05	6.00E-05	0.00016	0.00015
0.002	20,000	0.05	0.04874	0.03123	0.04853	**0.06676**	0.04437
		0.01	0.00972	0.00688	0.00979	**0.01447**	**0.01134**
		0.001	0.00097	8.00E-04	0.00101	**0.00191**	0.00111
		0.0001	0.00011	0.00016	0.00013	**0.00023**	0.00016
	30,000	0.05	0.04927	0.03057	0.04891	**0.06796**	0.04254
		0.01	0.00989	0.00650	0.00984	**0.01501**	**0.01366**
		0.001	0.00093	8.00E-04	0.00103	**0.00171**	**0.00156**
		0.0001	7.00E-05	0.00014	0.00012	**0.00023**	0.00015

Note: For significance levels 0.05, 0.01, 0.001, and 0.0001, and 100,000 replicates, the corresponding 95% confident intervals (CIs) are (0.04865, 0.05135), (0.00938, 0.01062), (0.0008, 0.0012), and (0.00004, 0.00016). Bold-faced values indicate the p-values beyond the upper bounds of the corresponding 95% CIs.

Table S2. The estimated type I error rates for two extremely case-control ratios (0.001 and 0.002) with two different sample sizes (20,000 and 30,000) under model 3 at different significant levels with 100,000 replicates.

Model 3			MLN-O	USAT	MANOVA	MultiPhen	TATES
C-C Ratio	Sample	α-level					
0.001	20,000	0.05	0.05065	0.03011	0.04829	**0.07587**	0.04311
		0.01	0.01036	0.00597	0.00953	**0.01775**	0.00863
		0.001	0.00097	0.00064	0.00084	**0.00199**	**0.00205**
		0.0001	9.00E-05	7.00E-05	6.00E-05	0.00019	1.00E-04
	30,000	0.05	0.04984	0.02955	0.04683	**0.05741**	0.04867
		0.01	0.00977	0.00599	0.00950	**0.01234**	0.01035
		0.001	0.00106	7.00E-04	0.00092	**0.00134**	0.00120
		0.0001	0.00011	5.00E-05	7.00E-05	9.00E-05	0.00017
0.002	20,000	0.05	0.04867	0.03069	0.04894	**0.06474**	0.04444
		0.01	0.01007	0.00661	0.00978	**0.01396**	**0.01146**
		0.001	0.00115	0.00065	0.00103	**0.00166**	**0.00142**
		0.0001	0.00011	5.00E-05	1.00E-04	0.00018	0.00016
	30,000	0.05	0.04980	0.02974	0.04826	**0.06656**	0.04265
		0.01	0.00951	0.00610	0.00951	**0.01461**	**0.0134**
		0.001	0.00096	0.00056	0.00083	**0.00164**	**0.00163**
		0.0001	1.00E-04	7.00E-05	7.00E-05	0.00015	0.00013

Note: For significance levels 0.05, 0.01, 0.001, and 0.0001 and 100,000 replicates, the corresponding 95% confident intervals (CIs) are (0.04865, 0.05135), (0.00938, 0.01062), (0.0008, 0.0012), and (0.00004, 0.00016). Bold-faced values indicate the p-values beyond the upper bounds of the corresponding 95% CIs.

Table S3. The estimated type I error rates for two extremely case-control ratios (0.001 and 0.002) with two different sample sizes (20,000 and 30,000) under model 4 at different significant levels with 100,000 replicates.

Model 4			MLN-O	USAT	MANOVA	MultiPhen	TATES
C-C Ratio	Sample	α-level					
0.001	20,000	0.05	0.04719	0.02926	0.04702	**0.07484**	0.04170
		0.01	0.00917	0.00593	0.00911	**0.01728**	0.00805
		0.001	0.00082	0.00059	0.00084	**0.00202**	**0.00168**
		0.0001	1.00E-04	2.00E-05	4.00E-05	0.00023	1.00E-04
	30,000	0.05	0.05053	0.02977	0.04975	**0.06844**	0.04272
		0.01	0.01009	0.00627	0.00961	**0.01469**	**0.01293**
		0.001	0.00110	0.00074	0.00101	**0.00156**	**0.00149**
		0.0001	9.00E-05	8.00E-05	0.00012	**0.00025**	7.00E-05
0.002	20,000	0.05	0.04814	0.03067	0.04892	**0.06571**	0.04336
		0.01	0.00985	0.00625	0.0095	**0.01398**	0.01070
		0.001	0.00076	0.00068	0.00086	**0.00151**	0.00115
		0.0001	1.00E-04	7.00E-05	1.00E-04	**0.00014**	0.00017
	30,000	0.05	0.04918	0.03083	0.04961	**0.06032**	0.05052
		0.01	0.00991	0.00672	0.01039	**0.01350**	0.01096
		0.001	0.00104	0.00073	0.00108	**0.00149**	0.00122
		0.0001	9.00E-05	1.00E-04	0.00011	**2.00E-04**	0.00016

Note: For significance levels0.05, 0.01, 0.001, and 0.0001, and 100,000 replicates, the corresponding 95% confident intervals (CIs) are (0.04865, 0.05135), (0.00938, 0.01062), (0.0008, 0.0012), (0.00004, 0.00016). Bold-faced values indicate the p-values beyond the upper bounds of the corresponding 95% CIs.

Table S4. The community detection results by the multi-layer network for the phenotypes in Chapter XIII (Diseases of the musculoskeletal system and connective tissue) in the UK Biobank.

Cluster (# phenotypes)	ICD10 code of phenotypes
I (5)	M720， M653， M674， M181， M189
II (2)	M796， M798
III (6)	M255， M232， M233， M238， M254， M659
IV (5)	M751， M754， M755， M750， M753
V (2)	M542， M758
VI (7)	M545， M512， M549， M543， M511， M544， M513
VII (4)	M503， M501， M500， M502
VIII (4)	M169， M199， M159， M139
IX (4)	M480， M419， M478， M479
X (2)	M202， M257
XI (4)	M179， M171， M170， M234
XII (2)	M161， M160
XIII (3)	M201， M204， M205
XIV (10)	M706， M678， M150， M109， M771， M533， M206， M224， M510， M130
XV (4)	M069， M819， M073， M858
XVI (2)	M350， M329
XVII (2)	M316， M353
XVIII (2)	M790， M797
XIX (2)	M45, M45X9

Table S5. The p-values estimated by all five methods for 321 SNPS detected only by MLN-O, the corresponding gene, and the references which reported the genes significantly associated with the phenotypes in Chapter XIII (Diseases of the musculoskeletal system and connective tissue) in the UK Biobank.

rsID	MLN.O	USAT	MANOVA	MultiOhen	TATES	Gene
Affx-28417083	4.28E-08	1.80E-05	2.51E-05	0.000153	0.000538	HLA-A[1, 2]
Affx-28441669	4.09E-09	1.01E-05	1.00E-05	1.78E-05	6.99E-08	C6orf15[3]; PSORS1C1[2-8]; CDSN
Affx-28446136	7.84E-11	1.25E-07	1.99E-07	3.38E-07	6.45E-05	HLA-C[5, 9]
Affx-52368556	1.14E-09	8.45E-07	1.08E-06	7.69E-07	0.0007	C6orf15; PSORS1C1; CDSN
rs1018430	1.73E-10	1.39E-06	1.27E-06	1.46E-06	0.001828	C6orf10
rs1018433	7.54E-10	3.01E-06	2.90E-06	2.71E-06	0.00717	C6orf10
rs1018434	1.43E-10	1.29E-06	1.26E-06	1.09E-06	0.003948	C6orf10
rs1041981	5.09E-12	1.17E-07	1.30E-07	1.58E-07	4.36E-05	NFKBIL1[10]; LTA[10]; TNF[7-9, 11-59]; LTB[24, 47, 53, 60-65]; LST1[8]; NCR3[47, 53, 66]
rs1042145	3.93E-09	1.33E-05	1.30E-05	2.29E-05	6.12E-08	C6orf15; PSORS1C1; CDSN
rs1045537	3.65E-09	1.35E-06	1.65E-06	1.74E-05	1.98E-07	HFE[67]; HIST1H4C; HIST1H1T; HIST1H2BC
rs10456362	4.84E-10	9.90E-05	0.000278	0.000382	0.00627	ZKSCAN4; NKAPL; ZSCAN26[10]
rs10484399	2.00E-12	7.62E-08	1.06E-07	1.23E-06	0.000112	NA
rs10484433	6.44E-09	1.75E-06	2.26E-06	2.49E-05	3.64E-07	HIST1H1A; HIST1H3A; HIST1H4A; HIST1H4B; HIST1H3B; HIST1H2AB; HIST1H2BB; HIST1H3C
rs10484435	3.31E-09	1.46E-06	1.98E-06	1.81E-05	7.79E-08	HIST1H1A; HIST1H3A; HIST1H4A; HIST1H4B; HIST1H3B; HIST1H2AB;

						HIST1H2BB; HIST1H3C
rs10484439	4.12E-11	1.93E-06	2.27E-06	2.40E-05	0.00024	NA
rs1049281	9.28E-11	2.48E-07	2.96E-07	5.71E-07	2.67E-05	HLA-C
rs1062630	1.83E-11	5.98E-07	7.62E-07	3.87E-07	6.87E-05	CCHCR1; TCF19; POU5F1[55, 68-70]
rs1065076	2.35E-11	1.57E-06	1.37E-06	5.14E-07	2.73E-06	MICB[71, 72]; MCCD1[2]
rs1076712	2.47E-08	0.001256	0.000687	0.000558	0.001824	C6orf10
rs1130838	1.22E-10	6.77E-07	7.45E-07	1.66E-06	7.46E-05	HLA-C
rs1150765	5.55E-11	3.31E-06	3.63E-06	1.33E-06	4.23E-05	PSORS1C1; CCHCR1; TCF19; POU5F1
rs11796	5.92E-12	2.20E-07	2.39E-07	2.53E-07	2.05E-05	MCCD1; DDX39B[71]; ATP6V1G2[71]; NFKBIL1
rs11965538	3.43E-10	3.60E-05	0.000258	0.0003	0.006059	ZKSCAN4; NKAPL; ZSCAN26; PGBD1[10]
rs12176317	1.02E-08	2.08E-05	1.99E-05	3.14E-05	0.004525	BTN3A2[68]; BTN2A2
rs1233396	1.61E-11	7.26E-08	1.17E-07	1.67E-07	0.000901	UBD; OR2H2[53]
rs1233478	1.74E-08	0.000205	0.000153	0.000227	0.015231	NA
rs1233480	1.60E-11	3.92E-07	6.18E-07	1.33E-06	0.002943	NA
rs1233489	1.13E-11	5.10E-07	6.90E-07	1.62E-06	0.000891	MAS1L
rs1233491	1.13E-11	1.62E-06	2.10E-06	3.17E-06	0.002306	MAS1L
rs1233493	4.49E-12	6.55E-07	8.88E-07	2.83E-06	0.001074	MAS1L
rs1233579	8.01E-13	6.08E-07	7.82E-07	2.59E-06	0.000274	NA
rs1233604	7.20E-11	3.64E-06	4.43E-06	1.05E-05	0.002219	NA
rs1233619	7.39E-13	1.08E-07	1.48E-07	4.58E-07	0.000233	NA
rs1265061	2.44E-08	9.26E-06	1.27E-05	1.28E-05	8.83E-06	C6orf15; PSORS1C1; CDSN
rs1265078	5.16E-10	2.86E-06	3.09E-06	1.41E-06	2.55E-07	PSORS1C1; PSORS1C2[4]; CCHCR1; TCF19; POU5F1
rs1265112	2.58E-10	3.74E-07	4.79E-07	2.16E-07	1.74E-07	PSORS1C1; PSORS1C2; CCHCR1; TCF19; POU5F1
rs1265114	3.24E-10	1.16E-06	1.37E-06	6.25E-07	1.52E-07	PSORS1C1; PSORS1C2; CCHCR1;

						TCF19; POU5F1
rs130071	7.53E-10	2.75E-05	2.77E-05	1.24E-05	6.87E-06	PSORS1C1; PSORS1C2; CCHCR1; TCF19; POU5F1
rs130076	2.22E-11	1.67E-07	1.89E-07	7.38E-08	4.95E-05	PSORS1C1; PSORS1C2; CCHCR1; TCF19; POU5F1
rs130079	1.17E-10	1.32E-06	1.48E-06	6.65E-07	5.40E-05	PSORS1C1; PSORS1C2; CCHCR1; TCF19; POU5F1
rs1311918	1.01E-12	1.69E-07	2.14E-07	8.19E-07	0.000228	NA
rs13194504	3.55E-13	7.85E-08	1.11E-07	6.61E-07	0.000496	NA
rs13194781	9.99E-12	3.61E-07	4.74E-07	2.99E-06	0.000315	HIST1H4K; HIST1H2BN; HIST1H2AK; HIST1H2AL; HIST1H1B
rs13195509	5.34E-09	1.19E-05	1.32E-05	1.31E-05	0.003617	BTN3A3; BTN2A1[43]
rs13196552	5.57E-09	1.05E-06	1.45E-06	1.61E-05	3.80E-07	TRIM38[73]
rs13198474	2.65E-08	3.96E-06	5.02E-06	3.06E-05	2.79E-05	SLC17A3[74]
rs13198716	7.95E-11	4.56E-07	6.02E-07	2.97E-06	0.001501	ABT1
rs13199772	1.10E-11	2.91E-07	3.73E-07	2.67E-06	0.000295	HIST1H2BN; HIST1H2AL; HIST1H1B; HIST1H3I; HIST1H4L
rs13200797	1.64E-08	2.77E-06	3.24E-06	2.04E-05	1.87E-07	HIST1H4C; HIST1H1T; HIST1H2BC; HIST1H2AC
rs13205911	8.31E-12	7.06E-08	9.65E-08	1.04E-06	0.000185	ZKSCAN8
rs13207673	6.73E-09	1.61E-06	2.23E-06	2.50E-05	6.13E-08	SLC17A2[8]
rs13212534	4.29E-09	3.22E-07	4.66E-07	7.07E-06	1.10E-05	TRIM38
rs1324087	3.69E-08	4.19E-06	4.25E-06	5.44E-05	1.39E-07	SLC17A1[29, 43, 44, 68, 75-78]; SLC17A3
rs1361385	2.36E-08	3.46E-06	0.001913	0.005382	0.003254	ZSCAN12
rs1416918	4.17E-08	4.48E-06	0.003348	0.008245	0.003118	ZSCAN12
rs1419183	3.79E-09	9.60E-05	0.000541	0.000521	0.011201	ZKSCAN4; NKAPL; ZSCAN26; PGBD1
rs14365	5.55E-10	6.27E-05	4.26E-05	5.20E-06	0.002274	BAG6[69]; APOM[79]; C6orf47;

						GPANK1; CSNK2B[80]; LY6G5B; LY6G5C; ABHD16A
rs149943	3.84E-10	8.41E-07	8.96E-07	5.03E-06	0.000835	NA
rs149990	6.79E-10	1.16E-06	1.12E-06	3.18E-06	0.000496	NA
rs1535039	7.05E-11	3.09E-06	4.03E-06	8.16E-06	0.001526	OR11A1; OR10C1; OR2H1
rs1558205	2.04E-08	8.06E-06	0.001551	0.004192	0.004503	ZSCAN12; ZSCAN23[8]
rs1559873	1.44E-10	1.30E-06	1.28E-06	1.37E-06	0.004524	C6orf10
rs1559874	7.77E-11	5.89E-07	6.27E-07	5.33E-07	0.00855	C6orf10
rs1576	5.83E-10	1.07E-06	1.19E-06	4.15E-07	1.11E-07	PSORS1C1; PSORS1C2; CCHCR1; TCF19
rs1632854	1.25E-08	0.00014	0.00014	0.000145	0.000495	MUC21[2, 8, 30, 43]; MUC22[2, 8, 43, 58, 69]
rs1679709	6.06E-10	6.08E-05	0.000334	0.000395	0.005921	ZKSCAN4; NKAPL; ZSCAN26
rs16891264	1.94E-09	1.23E-06	1.80E-06	1.65E-05	1.17E-07	HIST1H1C; HFE
rs16891315	2.25E-09	2.44E-07	3.28E-07	4.17E-06	7.20E-07	HFE; HIST1H4C; HIST1H1T; HIST1H2BC; HIST1H2AC
rs16891334	1.95E-09	7.12E-07	8.98E-07	8.45E-06	3.06E-07	HIST1H4C; HIST1H1T; HIST1H2BC; HIST1H2AC
rs16894060	1.21E-08	4.31E-06	0.001646	0.005013	0.00471	ZSCAN12
rs17533090	5.89E-09	6.36E-06	4.31E-06	6.94E-06	8.54E-06	HLA-DQA1[1, 2, 5, 7, 8, 15, 16, 21, 25, 30, 36, 53, 56, 58, 68, 69, 80-94]
rs175597	2.01E-12	2.53E-07	3.24E-07	1.76E-06	5.36E-05	HIST1H4J; HIST1H4K; HIST1H2BN; HIST1H2AK
rs17693963	4.74E-11	2.51E-06	3.23E-06	1.92E-05	0.000248	NA
rs17720293	4.15E-12	9.19E-06	1.17E-05	2.52E-05	0.001211	ZSCAN9; ZKSCAN4; NKAPL
rs17749927	1.19E-11	1.16E-06	1.45E-06	7.35E-06	0.00029	NA
rs1778508	9.24E-10	6.12E-05	0.000415	0.000467	0.005809	ZKSCAN4; NKAPL; ZSCAN26; PGBD1

rs1977	3.49E-08	0.000111	9.95E-05	0.000175	0.006684	BTN3A2; BTN2A2
rs1979	4.38E-09	1.54E-05	1.48E-05	2.18E-05	0.004536	BTN3A2; BTN2A2
rs200482	1.51E-12	2.67E-07	3.50E-07	1.86E-06	5.77E-05	HIST1H2BL; HIST1H2AI; HIST1H3H; HIST1H2AJ; HIST1H2BM; HIST1H4J
rs200483	2.08E-12	8.17E-07	9.72E-07	4.75E-06	6.96E-05	HIST1H2BL; HIST1H2AI; HIST1H3H; HIST1H2AJ; HIST1H2BM; HIST1H4J
rs200490	3.91E-12	2.86E-07	3.70E-07	2.46E-06	4.87E-05	HIST1H3H; HIST1H2AJ; HIST1H2BM; HIST1H4J; HIST1H4K; HIST1H2BN; HIST1H2AK
rs200953	5.06E-12	3.47E-07	4.45E-07	2.55E-06	5.09E-05	HIST1H2BN; HIST1H2AL; HIST1H1B; HIST1H3I; HIST1H4L
rs200956	9.49E-12	2.29E-05	3.08E-05	4.07E-05	0.001131	HIST1H2BN; HIST1H2AL; HIST1H1B; HIST1H3I; HIST1H4L; HIST1H3J
rs200991	1.99E-11	2.67E-05	3.78E-05	6.68E-05	0.000399	HIST1H4K; HIST1H2BN; HIST1H2AK; HIST1H2AL; HIST1H1B
rs200995	1.74E-12	3.27E-07	4.27E-07	2.10E-06	5.38E-05	HIST1H4K; HIST1H2BN; HIST1H2AK; HIST1H2AL
rs201002	6.34E-12	8.46E-07	1.03E-06	5.99E-06	6.38E-05	HIST1H4J; HIST1H4K; HIST1H2BN; HIST1H2AK
rs201004	2.90E-08	0.002061	0.001424	0.001215	0.004807	HIST1H4J; HIST1H4K; HIST1H2BN; HIST1H2AK
rs2022083	7.48E-10	2.21E-07	2.67E-07	6.74E-07	0.000381	NA
rs202906	6.71E-10	1.46E-06	1.63E-06	6.53E-06	0.000523	NA
rs2071591	1.09E-11	2.04E-07	2.26E-07	1.78E-07	3.46E-05	MCCD1; DDX39B;

						ATP6V1G2; NFKBIL1
rs2071594	1.12E-11	2.32E-07	2.50E-07	2.32E-07	1.47E-05	MCCD1; DDX39B; ATP6V1G2; NFKBIL1
rs2071965	1.71E-08	2.50E-06	0.002161	0.007245	0.002188	ZSCAN23
rs2072803	4.93E-08	2.81E-05	2.91E-05	2.12E-05	0.008766	BTN3A2; BTN2A2; BTN3A1
rs213230	3.91E-08	1.19E-05	0.001723	0.005905	0.007249	ZSCAN31[68]; ZKSCAN3; ZSCAN12
rs2233956	7.77E-11	7.15E-05	7.40E-05	0.00011	6.03E-06	C6orf15; PSORS1C1; CDSN
rs2239527	7.40E-13	1.23E-07	1.49E-07	1.91E-07	1.16E-05	MCCD1; DDX39B; ATP6V1G2; NFKBIL1
rs2239529	2.22E-09	4.43E-07	6.49E-07	1.72E-06	0.000172	TRIM31[2, 7, 69]
rs2242653	2.29E-11	5.08E-06	4.83E-06	1.86E-06	0.000793	ABHD16A; LY6G6F; LY6G6D; LY6G6C; C6orf25; DDAH2
rs2242655	2.13E-13	1.20E-07	1.22E-07	5.60E-08	2.67E-05	BAG6; APOM; C6orf47; GPANK1; CSNK2B; LY6G5B; LY6G5C
rs2243868	1.24E-12	1.17E-07	1.37E-07	1.96E-07	8.65E-08	NA
rs2249099	3.89E-08	1.54E-05	1.90E-05	2.71E-05	0.00041	TRIM31
rs2280800	1.04E-13	1.24E-07	1.27E-07	5.51E-08	2.82E-05	C6orf47; GPANK1; CSNK2B; LY6G5B; LY6G5C; ABHD16A
rs2295664	3.04E-13	1.58E-07	1.59E-07	6.28E-08	2.50E-05	BAG6; APOM; C6orf47; GPANK1; CSNK2B; LY6G5B; LY6G5C
rs2295665	7.05E-13	3.68E-07	3.65E-07	9.17E-08	0.000167	BAG6; APOM; C6orf47; GPANK1; CSNK2B;

						LY6G5B; LY6G5C
rs2394894	9.56E-12	4.89E-07	6.48E-07	5.24E-07	0.000179	NA
rs2394895	1.61E-11	1.42E-06	1.70E-06	1.35E-06	0.000176	NA
rs2395045	4.03E-09	0.000501	0.000264	0.000139	1.87E-05	MICB; MCCD1; DDX39B
rs2442752	3.82E-09	1.04E-05	8.62E-06	1.31E-05	1.07E-07	MICA[2, 5, 7, 8, 43, 47, 60, 89, 95]
rs2507997	5.89E-09	5.34E-07	5.31E-07	8.69E-07	5.72E-05	HLA-B[2, 8, 43, 49, 53, 58, 96, 97]
rs2516491	1.56E-12	2.79E-07	3.70E-07	1.06E-06	4.45E-07	MICB; MCCD1; DDX39B
rs2517403	7.74E-09	8.54E-06	1.22E-05	1.23E-05	2.61E-06	C6orf15; PSORS1C1; CDSN
rs2517485	8.31E-10	1.62E-05	2.23E-05	1.98E-05	4.25E-06	C6orf15; PSORS1C1; CDSN
rs2517544	9.47E-09	6.86E-06	8.11E-06	1.70E-05	0.000505	MUC22
rs2517611	1.50E-08	1.43E-07	2.45E-07	5.71E-07	0.007255	TRIM26[69]
rs2517613	1.17E-10	6.09E-08	8.31E-08	2.69E-07	0.005079	TRIM26
rs2517645	4.75E-09	9.73E-07	1.26E-06	9.01E-07	0.002588	TRIM40; TRIM10; TRIM15
rs2523721	1.61E-08	4.09E-07	6.54E-07	7.98E-07	0.008966	TRIM26
rs2523722	6.69E-09	8.18E-08	1.46E-07	2.87E-07	0.005499	TRIM26
rs2523933	7.36E-09	6.99E-05	3.53E-05	6.50E-05	0.022604	HLA-A
rs2523986	1.33E-10	1.54E-07	2.40E-07	1.18E-06	8.11E-05	TRIM31
rs2523989	1.17E-08	3.76E-06	4.92E-06	9.14E-06	0.000237	TRIM31
rs2524074	2.70E-11	1.58E-07	1.90E-07	2.97E-07	2.00E-05	HLA-C
rs2524084	4.81E-08	6.00E-05	8.40E-05	3.51E-05	3.44E-05	HLA-C
rs2524089	3.70E-12	8.34E-08	9.87E-08	1.35E-07	5.75E-08	NA
rs2524156	1.25E-10	9.97E-07	1.06E-06	1.47E-06	1.91E-07	NA
rs2524163	1.13E-12	5.03E-08	5.94E-08	8.57E-08	6.64E-08	HLA-C
rs2596472	1.39E-10	3.85E-05	2.80E-05	3.63E-05	2.81E-07	NA
rs2734573	2.35E-12	1.71E-06	1.43E-06	5.06E-07	7.79E-07	MICB; MCCD1; DDX39B; ATP6V1G2; NFKBIL1
rs2734574	2.69E-10	3.84E-05	3.26E-05	1.77E-05	0.000634	MICB; MCCD1; DDX39B; ATP6V1G2
rs2746149	3.95E-12	5.87E-07	7.99E-07	2.73E-06	0.001108	OR11A1; OR2H1; MAS1L

rs2746150	3.51E-12	5.41E-07	7.53E-07	1.73E-06	0.000829	OR11A1; OR2H1; MAS1L
rs2799077	2.79E-09	9.80E-05	0.00109	0.001539	0.007754	ZKSCAN4; NKAPL; ZSCAN26; PGBD1
rs2799079	4.17E-10	3.77E-05	0.000209	0.000221	0.012117	ZKSCAN4; NKAPL; ZSCAN26; PGBD1
rs28366353	1.64E-08	7.15E-07	6.41E-07	3.85E-07	1.61E-07	HLA-DRB1[1, 2, 5, 7, 8, 15, 16, 21, 25, 29, 36, 49, 53, 56, 58, 81, 82, 85, 86, 89, 90, 93]
rs28367630	5.98E-09	6.99E-06	4.96E-06	1.05E-05	5.62E-08	NA
rs2844575	8.80E-13	4.32E-06	4.79E-06	1.09E-05	1.22E-07	HLA-B
rs2844635	2.08E-08	1.37E-05	1.82E-05	1.58E-05	4.20E-06	C6orf15; PSORS1C1; CDSN
rs2844645	1.26E-08	0.000963	0.000565	0.000754	1.28E-05	MUC22
rs2844647	2.89E-08	0.00152	0.000875	0.001395	8.14E-05	MUC22
rs2844773	4.13E-10	7.97E-08	1.02E-07	3.24E-07	0.002136	NA
rs2844793	5.39E-09	1.66E-06	2.43E-06	3.14E-06	0.00023	TRIM31
rs2858332	2.63E-08	2.49E-07	2.59E-07	2.06E-07	0.004342	NA
rs2894239	2.97E-08	4.36E-07	4.51E-07	7.79E-07	1.41E-05	NA
rs2894240	1.50E-08	5.79E-07	5.87E-07	1.01E-06	1.31E-05	NA
rs3093974	1.45E-12	1.22E-07	1.41E-07	1.12E-07	1.02E-05	MCCD1; DDX39B; ATP6V1G2; NFKBIL1
rs3093999	2.91E-09	0.00146	0.000765	0.000504	5.08E-06	MICB; MCCD1; DDX39B
rs3094070	4.77E-10	5.51E-08	6.50E-08	1.67E-07	0.000658	NA
rs3094071	8.54E-10	7.26E-08	8.69E-08	2.88E-07	0.000951	NA
rs3094122	6.57E-09	8.24E-08	1.07E-07	2.05E-07	5.07E-06	FLOT1[98]; IER3
rs3094212	6.80E-09	1.52E-05	1.49E-05	3.53E-05	5.81E-08	C6orf15; PSORS1C1; CDSN; PSORS1C2
rs3094215	4.74E-09	1.18E-05	1.15E-05	3.01E-05	6.49E-08	C6orf15; PSORS1C1; CDSN
rs3094216	2.67E-09	6.94E-07	8.51E-07	4.47E-07	0.000431	C6orf15; PSORS1C1; CDSN
rs3094220	1.53E-09	3.38E-07	4.27E-07	2.16E-07	0.000433	C6orf15; PSORS1C1; CDSN

rs3094672	7.07E-09	0.000566	0.000302	0.001632	0.000231	MUC22
rs3094694	2.85E-09	1.88E-06	3.86E-06	1.22E-05	2.35E-06	HLA-E
rs3095298	1.87E-09	5.07E-07	6.31E-07	3.12E-07	0.000526	C6orf15; PSORS1C1; CDSN
rs3095340	2.06E-11	5.07E-08	8.78E-08	2.06E-07	7.63E-06	FLOT1; IER3
rs3096673	1.76E-10	1.97E-06	1.79E-06	1.59E-06	0.001663	NA
rs3096674	1.24E-09	8.21E-06	7.49E-06	8.68E-06	0.012472	NA
rs3096681	2.57E-10	1.35E-06	1.23E-06	1.34E-06	0.001633	NA
rs3115553	2.19E-10	1.74E-06	1.61E-06	1.86E-06	0.001563	C6orf10
rs3115560	4.14E-10	2.00E-06	1.86E-06	1.89E-06	0.00244	NA
rs3115563	7.24E-10	1.93E-06	1.74E-06	1.77E-06	0.001898	NA
rs3115569	2.63E-10	3.15E-06	2.82E-06	2.67E-06	0.001893	NA
rs3115573	1.75E-08	3.42E-07	3.58E-07	6.20E-07	1.28E-05	NA
rs3116830	3.02E-13	1.07E-07	1.31E-07	1.13E-06	0.000142	NA
rs3117122	2.87E-08	0.001546	0.00094	0.000981	0.001277	C6orf10
rs3117143	6.98E-13	1.40E-07	1.67E-07	1.47E-06	0.000162	OR2W1
rs3118359	1.10E-12	1.31E-07	1.61E-07	7.74E-07	0.000223	NA
rs3118361	1.05E-12	2.02E-07	2.33E-07	7.25E-07	0.000847	TRIM27[47]
rs3129788	1.45E-12	3.92E-07	4.41E-07	1.37E-06	0.000395	OR2B3[5]
rs3129791	7.54E-13	2.94E-07	3.72E-07	1.57E-06	0.000321	ZNF311
rs3129831	2.55E-09	1.09E-07	1.23E-07	3.83E-07	0.003342	NA
rs3129832	5.08E-10	6.07E-08	7.83E-08	2.06E-07	0.002661	NA
rs3130059	2.58E-12	5.41E-08	6.07E-08	5.08E-08	1.64E-05	MCCD1; DDX39B; ATP6V1G2; NFKBIL1
rs3130315	8.95E-09	2.18E-07	2.32E-07	4.20E-07	1.01E-05	NA
rs3130320	1.92E-09	1.23E-07	1.14E-07	1.90E-07	2.92E-06	NA
rs3130340	2.09E-10	6.28E-07	5.69E-07	5.82E-07	0.001404	C6orf10
rs3130424	1.29E-11	8.87E-07	1.14E-06	9.10E-07	0.00017	HLA-C
rs3130455	1.31E-11	3.16E-07	3.75E-07	2.53E-07	0.000119	PSORS1C1; PSORS1C2; CCHCR1; TCF19; POU5F1
rs3130457	1.28E-11	5.65E-07	6.48E-07	3.16E-07	5.55E-05	TCF19; POU5F1
rs3130467	2.60E-11	4.92E-07	5.43E-07	8.45E-07	2.00E-05	NA
rs3130473	7.22E-12	2.79E-07	3.43E-07	3.92E-07	2.58E-05	NA
rs3130517	1.96E-11	4.20E-07	5.08E-07	7.64E-07	2.90E-05	NA
rs3130555	6.78E-09	1.99E-05	1.93E-05	3.35E-05	6.38E-08	C6orf15; PSORS1C1; CDSN; PSORS1C2
rs3130615	3.35E-11	2.26E-06	1.96E-06	9.55E-07	3.00E-06	MICB
rs3130662	8.69E-11	1.25E-07	1.58E-07	5.31E-07	0.000317	FLOT1; IER3

rs3130713	6.14E-12	3.16E-07	3.87E-07	5.23E-07	4.93E-05	NA
rs3130834	1.85E-13	5.17E-08	7.03E-08	3.77E-07	0.000149	NA
rs3130837	1.79E-12	3.60E-07	4.15E-07	1.79E-06	0.000341	ZNF311
rs3130838	1.28E-12	2.04E-07	2.63E-07	1.12E-06	0.001146	TRIM27
rs3130845	6.65E-13	1.03E-07	1.33E-07	6.15E-07	0.000379	NA
rs3130893	1.04E-12	2.51E-07	2.95E-07	1.05E-06	0.000343	ZNF311
rs3130942	1.20E-11	1.14E-06	1.35E-06	1.56E-06	4.90E-05	NA
rs3130944	1.31E-11	1.03E-06	1.27E-06	1.30E-06	4.94E-05	NA
rs3130953	2.57E-11	4.71E-07	5.40E-07	8.96E-07	3.12E-05	NA
rs3130977	2.20E-08	3.00E-06	3.17E-06	3.50E-06	0.000461	C6orf15; PSORS1C1; CDSN
rs3131018	2.16E-08	0.001182	0.000572	0.00101	6.49E-07	CCHCR1; TCF19; POU5F1
rs3131064	2.68E-09	6.72E-06	7.66E-06	9.41E-06	0.003796	NA
rs3131073	4.11E-13	1.15E-07	1.43E-07	1.28E-06	0.000291	NA
rs3131343	1.97E-12	1.54E-07	1.88E-07	7.89E-07	0.00027	NA
rs3131631	3.65E-09	0.000453	0.000238	0.000133	1.56E-05	MICB; MCCD1; DDX39B
rs3131635	4.14E-11	2.01E-06	1.74E-06	5.56E-07	3.23E-06	MICB
rs3131638	2.69E-10	1.97E-06	1.75E-06	5.26E-07	6.86E-05	MICB
rs3132390	8.95E-12	2.63E-07	3.33E-07	1.44E-06	0.000962	NA
rs3132392	3.31E-12	2.37E-07	2.81E-07	1.51E-06	0.000323	NA
rs3132468	4.19E-11	1.74E-06	1.51E-06	7.37E-07	2.87E-06	MICB
rs3132505	4.52E-12	6.60E-07	8.60E-07	9.27E-07	0.00023	NA
rs3132506	1.13E-11	1.08E-06	1.46E-06	1.06E-06	0.00015	NA
rs3132550	2.07E-09	4.42E-07	5.48E-07	3.07E-07	0.000389	C6orf15; PSORS1C1; CDSN; PSORS1C2
rs3132554	3.78E-09	1.04E-05	1.04E-05	1.86E-05	6.18E-08	C6orf15; PSORS1C1; CDSN
rs3132928	6.74E-10	3.57E-06	3.40E-06	3.66E-06	0.00355	NA
rs3132931	2.80E-10	1.84E-06	1.83E-06	1.54E-06	0.007761	NA
rs3134762	1.23E-11	1.02E-06	1.29E-06	1.17E-06	0.000179	NA
rs3134899	5.78E-09	2.42E-05	1.88E-05	8.16E-06	0.000131	MICB
rs34333163	1.68E-08	1.23E-06	2.26E-05	0.000175	0.02852	SLC39A8[1, 56, 63, 65, 99, 100]
rs34411532	3.69E-09	4.39E-06	3.05E-06	6.20E-06	4.60E-06	HLA-DQA1
rs34525648	5.08E-09	3.22E-06	4.41E-06	7.67E-05	5.58E-08	SLC17A2
rs34706883	3.81E-12	2.54E-07	3.38E-07	2.24E-06	0.000336	HIST1H4J; HIST1H4K; HIST1H2BN; HIST1H2AK

rs34788973	4.13E-09	4.20E-06	4.49E-06	5.15E-06	0.014194	HIST1H2AM; HIST1H2BO; OR2B2[10]
rs35555795	1.01E-09	6.79E-06	7.56E-06	9.80E-06	0.003406	BTN1A1
rs370155	2.68E-12	4.22E-07	5.47E-07	2.22E-06	0.000113	HIST1H2BL; HIST1H2AI; HIST1H3H; HIST1H2AJ; HIST1H2BM; HIST1H4J; HIST1H4K
rs3734523	8.04E-09	6.63E-06	8.70E-06	0.000146	5.13E-08	SLC17A2
rs3734542	2.81E-09	1.21E-05	1.35E-05	1.82E-05	0.007224	BTN3A3; BTN2A1
rs3734543	2.36E-09	7.36E-06	7.71E-06	1.62E-05	0.002043	BTN3A3; BTN2A1
rs3734563	2.38E-08	4.43E-06	0.002798	0.008121	0.003642	ZKSCAN3; ZSCAN12
rs3749953	1.11E-08	0.006528	0.003537	0.003555	8.67E-05	C6orf25; DDAH2; CLIC1; MSH5[5, 80, 101]; SAPCD1[5, 80, 101]
rs3749966	1.61E-10	1.15E-06	1.05E-06	1.01E-06	0.0013	C6orf10
rs3749971	1.22E-12	1.69E-07	2.50E-07	3.93E-07	0.000608	OR5V1[68]; OR12D3
rs3757188	8.92E-10	9.42E-05	0.000125	0.000243	0.001217	ZSCAN16[53]; ZKSCAN8
rs3799383	2.77E-08	5.42E-05	8.28E-05	4.48E-05	0.025854	BTN1A1
rs3800328	1.80E-08	2.97E-06	0.002189	0.006135	0.002547	ZSCAN23
rs3864300	5.83E-10	3.31E-06	3.13E-06	2.97E-06	0.010385	C6orf10
rs3864302	5.64E-10	4.19E-06	3.59E-06	3.28E-06	0.002145	C6orf10
rs404240	2.11E-12	1.19E-07	1.76E-07	4.24E-07	0.000393	UBD
rs41266839	2.81E-11	1.91E-07	2.71E-07	1.72E-06	0.00116	BTN2A2; BTN3A1
rs4254981	2.85E-08	4.64E-06	0.003528	0.009168	0.003699	ZSCAN12
rs429479	1.30E-12	2.24E-07	3.25E-07	8.18E-07	0.002008	OR12D2
rs4324798	1.47E-12	2.00E-07	2.44E-07	1.25E-06	0.000511	NA
rs4357130	1.65E-08	2.19E-06	0.001547	0.004705	0.002647	ZSCAN12
rs4394275	4.71E-08	1.15E-05	1.10E-05	0.000112	0.0003	HLA-B
rs442694	1.48E-11	1.06E-06	1.41E-06	3.28E-06	0.000773	OR12D3; OR12D2
rs4634439	5.41E-09	8.91E-06	1.07E-05	1.84E-05	0.00796	ABT1
rs4713139	2.81E-10	3.93E-05	3.95E-05	7.11E-05	0.001486	ZSCAN16; ZKSCAN8
rs4713140	2.10E-09	7.63E-05	0.000358	0.000321	0.018107	ZSCAN16; ZKSCAN8
rs4947350	5.08E-09	2.63E-05	2.61E-05	4.62E-05	4.99E-06	HLA-DOB[5, 10, 49, 102, 103]

rs6457536	2.12E-10	8.44E-07	8.39E-07	1.20E-06	0.006201	C6orf10
rs68141011	2.53E-09	0.000245	0.000487	0.000863	0.005051	ZSCAN9; ZKSCAN4; NKAPL; ZSCAN26
rs6899389	3.22E-08	2.96E-06	0.003091	0.007828	0.002552	ZSCAN12; ZSCAN23
rs6901575	1.19E-09	9.75E-05	0.000361	0.00037	0.005568	ZSCAN26; PGBD1
rs6907950	1.53E-08	2.38E-06	0.001998	0.005368	0.002514	ZSCAN12
rs6908137	3.53E-08	4.01E-06	0.002746	0.007397	0.003385	ZSCAN12
rs6908726	7.45E-12	6.03E-07	8.36E-07	3.04E-06	0.000447	NA
rs6909427	1.19E-10	4.85E-07	4.63E-07	5.94E-07	0.001987	C6orf10
rs6909790	5.78E-10	2.12E-06	2.00E-06	1.74E-06	0.006784	C6orf10
rs6915455	4.59E-10	1.60E-06	1.57E-06	1.47E-06	0.00627	C6orf10
rs6922169	3.30E-08	5.79E-06	0.002653	0.007268	0.004472	ZSCAN12; ZSCAN23
rs6934429	5.45E-10	2.16E-06	2.05E-06	2.04E-06	0.007077	C6orf10
rs6934776	1.83E-10	1.38E-06	1.36E-06	1.38E-06	0.004707	C6orf10
rs6935269	4.75E-10	2.26E-06	2.07E-06	2.62E-06	0.002002	C6orf10
rs72841509	2.19E-08	5.23E-05	4.83E-05	7.45E-05	0.005477	BTN3A2; BTN2A2
rs7341328	2.95E-09	7.11E-06	6.52E-06	3.98E-06	0.009163	C6orf10
rs735765	2.70E-10	2.13E-05	5.41E-05	5.55E-05	0.002492	NA
rs746647	3.78E-10	1.48E-06	1.66E-06	6.99E-07	1.65E-07	PSORS1C1; PSORS1C2; CCHCR1; TCF19; POU5F1
rs761188	4.71E-08	0.002012	0.001161	0.000806	0.001911	C6orf10
rs7742654	1.47E-09	2.46E-05	2.15E-05	3.01E-05	0.006213	C6orf10
rs7748167	6.07E-09	2.16E-05	2.66E-05	0.000246	1.10E-07	SLC17A2
rs7751896	3.75E-10	2.28E-06	2.04E-06	1.89E-06	0.002906	C6orf10
rs7764722	2.59E-08	3.50E-06	0.002037	0.005488	0.003292	ZKSCAN3; ZSCAN12
rs7767099	2.50E-12	2.79E-07	3.73E-07	1.47E-06	0.000484	NA
rs7767325	2.52E-10	2.04E-06	1.88E-06	1.93E-06	0.001944	NA
rs7773051	2.32E-08	5.23E-06	0.002888	0.00776	0.004561	ZSCAN31; ZKSCAN3; ZSCAN12
rs7774434	3.56E-08	6.28E-06	4.69E-06	8.70E-06	0.000664	NA
rs853676	2.06E-09	3.83E-05	0.000268	0.000197	0.013072	ZSCAN31; ZKSCAN3
rs853678	1.25E-09	2.29E-05	0.000234	0.00016	0.011501	ZSCAN31
rs853679	2.81E-09	3.07E-05	0.000343	0.000192	0.017338	ZSCAN31
rs887465	6.75E-12	5.25E-07	6.40E-07	2.40E-07	4.97E-05	CCHCR1; TCF19; POU5F1

rs887468	5.95E-09	1.41E-05	1.56E-05	1.52E-05	0.000366	CCHCR1; TCF19; POU5F1
rs909253	1.97E-12	1.51E-07	1.62E-07	1.76E-07	2.82E-05	NFKBIL1; LTA; TNF; LTB; LST1; NCR3
rs915664	4.07E-08	1.10E-05	9.47E-06	1.98E-05	2.12E-07	NA
rs9257248	1.19E-12	1.47E-07	1.78E-07	8.75E-07	0.000514	NA
rs9257793	5.33E-10	3.02E-06	4.02E-06	1.66E-05	0.001867	OR5V1; OR12D3
rs9257802	2.75E-09	0.000132	0.000121	9.39E-05	0.030964	OR5V1; OR12D3
rs9257805	3.64E-12	9.68E-08	1.61E-07	4.88E-07	0.001598	OR12D3; OR12D2
rs9257809	3.50E-12	4.10E-07	5.86E-07	1.16E-06	0.000727	OR12D3; OR12D2
rs9263708	2.33E-09	3.30E-07	4.27E-07	3.51E-07	0.000382	C6orf15; PSORS1C1; CDSN; PSORS1C2; CCHCR1
rs9264643	3.88E-11	4.04E-07	4.71E-07	1.18E-06	3.79E-05	HLA-C
rs926552	2.08E-11	1.01E-07	1.55E-07	2.25E-07	0.001119	OR2H2
rs9265604	7.71E-11	3.86E-06	4.72E-06	4.56E-06	5.95E-06	NA
rs9267971	1.55E-08	2.38E-07	2.54E-07	5.00E-07	1.86E-05	NA
rs9268055	2.23E-10	2.06E-06	1.84E-06	2.15E-06	0.001546	NA
rs9268135	7.52E-11	1.33E-06	1.26E-06	2.67E-06	0.001911	C6orf10
rs9268137	1.23E-09	9.39E-06	8.46E-06	8.46E-06	0.011231	C6orf10
rs9268165	7.87E-10	8.34E-06	7.83E-06	6.64E-06	0.008758	C6orf10
rs9268176	1.95E-10	1.71E-06	1.69E-06	1.54E-06	0.00536	C6orf10
rs9268197	3.63E-10	3.30E-06	3.22E-06	2.93E-06	0.008026	C6orf10
rs9268199	2.61E-08	5.08E-05	4.37E-05	1.48E-05	1.11E-07	C6orf10
rs9268202	1.80E-10	2.11E-06	2.10E-06	1.80E-06	0.005338	C6orf10
rs9268212	2.17E-10	1.79E-06	1.76E-06	1.41E-06	0.006636	C6orf10
rs9268213	1.95E-09	3.62E-06	3.49E-06	2.52E-06	0.0084	C6orf10
rs9273364	1.26E-12	6.67E-08	9.22E-08	1.62E-07	0.000139	HLA-DQA1; HLA-DQB1[1, 8, 10, 17, 49, 58, 61, 71, 80, 81, 84, 87, 88, 94, 104-107]
rs9295740	1.56E-08	0.003192	0.002329	0.002503	0.004149	NA
rs9295768	1.69E-11	6.98E-06	7.72E-06	1.09E-05	0.004694	ZSCAN9; ZKSCAN4; NKAPL
rs9358934	3.44E-08	7.18E-05	8.15E-05	8.01E-05	0.008073	BTN3A2; BTN2A2
rs9379855	1.08E-08	3.27E-05	3.00E-05	4.24E-05	0.005952	BTN3A2; BTN2A2

rs9379858	1.94E-08	5.04E-05	4.55E-05	6.61E-05	0.006013	BTN3A2; BTN2A2
rs9379897	4.74E-09	1.35E-05	1.58E-05	2.79E-05	0.008005	ABT1
rs9380064	4.91E-10	5.88E-05	7.11E-05	0.000132	0.002669	ZKSCAN8
rs9380069	7.25E-10	5.45E-05	5.33E-05	8.03E-05	0.001533	ZSCAN9; ZKSCAN4
rs9393705	1.58E-08	2.96E-05	2.70E-05	5.72E-05	0.004567	BTN3A2
rs9393708	1.81E-08	3.41E-05	3.21E-05	4.41E-05	0.005919	BTN3A2
rs9393713	7.94E-09	2.71E-05	2.52E-05	4.86E-05	0.004393	BTN3A2; BTN2A2
rs9393714	6.39E-09	1.90E-05	1.84E-05	3.32E-05	0.004316	BTN3A2; BTN2A2
rs9393909	7.25E-12	7.70E-06	9.11E-06	1.30E-05	0.003724	ZSCAN9; ZKSCAN4; NKAPL
rs9393929	2.82E-11	4.11E-07	5.38E-07	1.26E-06	0.001201	NA
rs9461458	4.01E-08	3.92E-06	0.002668	0.006646	0.003184	ZKSCAN3; ZSCAN12
rs9468287	1.50E-10	1.11E-05	1.12E-05	1.63E-05	0.001061	ZSCAN16
rs9468317	2.71E-12	3.89E-06	4.57E-06	6.51E-06	0.002772	ZSCAN9; ZKSCAN4
rs9468370	2.81E-08	3.00E-06	0.002798	0.007119	0.002793	ZSCAN12
rs967005	1.28E-11	5.31E-06	5.97E-06	6.90E-06	0.00337	ZSCAN9; ZKSCAN4; NKAPL
rs971570	4.02E-09	5.57E-08	9.34E-08	1.20E-07	0.006592	TRIM26

Note: NA means the corresponding SNP does not have a mapped gene. There are 50 genes in the last column with superscripts which represent the order of references. As out best knowledge, the references listed in the table also reported the corresponding gene is correlated with the diseases of the musculoskeletal system and connective tissue. if a set of SNPs map to the same gene, only the first gene has a superscript.

Table S6. The estimated type I error rates for two extremely case-control ratios (0.001 and 0.002) with two different sample sizes (20,000 and 30,000) under model 5 at different significant levels with 100,000 replicates.

	Model 5		MLN-O	USAT	MANOVA	MultiPhen	TATES
C-C Ratio	Sample	α-level					
0.001	20,000	0.05	0.04986	0.03032	0.04752	**0.0765**	0.04203
		0.01	0.01006	0.00611	0.00926	**0.01732**	0.00834
		0.001	0.00103	0.00062	9.00E-04	**0.00201**	**0.00196**
		0.0001	6.00E-05	6.00E-05	6.00E-05	**0.00025**	**0.00016**
	30,000	0.05	0.04847	0.03149	0.04771	**0.06885**	0.04332
		0.01	0.00995	0.00664	0.00947	**0.01531**	0.01088
		0.001	0.00101	0.00072	0.00079	**0.00177**	0.00119
		0.0001	0.00013	0.00008	7.00E-05	**0.00024**	**0.00018**
0.002	20,000	0.05	0.04874	0.02939	0.04825	**0.06712**	0.04345
		0.01	0.00918	0.00606	0.00941	**0.01463**	**0.01428**
		0.001	0.00091	0.00063	0.001	**0.00184**	**0.00134**
		0.0001	1.00E-04	0.00009	0.00012	**0.00024**	0.00012
	30,000	0.05	0.05036	0.02962	0.04876	**0.06089**	0.04837
		0.01	0.01018	0.00605	0.00962	**0.01346**	0.01038
		0.001	0.0012	0.00065	0.00108	**0.00155**	0.00115
		0.0001	0.00012	0.00002	7.00E-05	**0.00011**	**0.00016**

Note: For significance levels 0.05, 0.01, 0.001, and 0.0001, and 100,000 replicates, the corresponding 95% confident intervals (CIs) are (0.04865, 0.05135), (0.00938, 0.01062), (0.0008, 0.0012), and (0.00004, 0.00016). Bold-faced values indicate the p-values beyond the upper bounds of the corresponding 95% CIs.

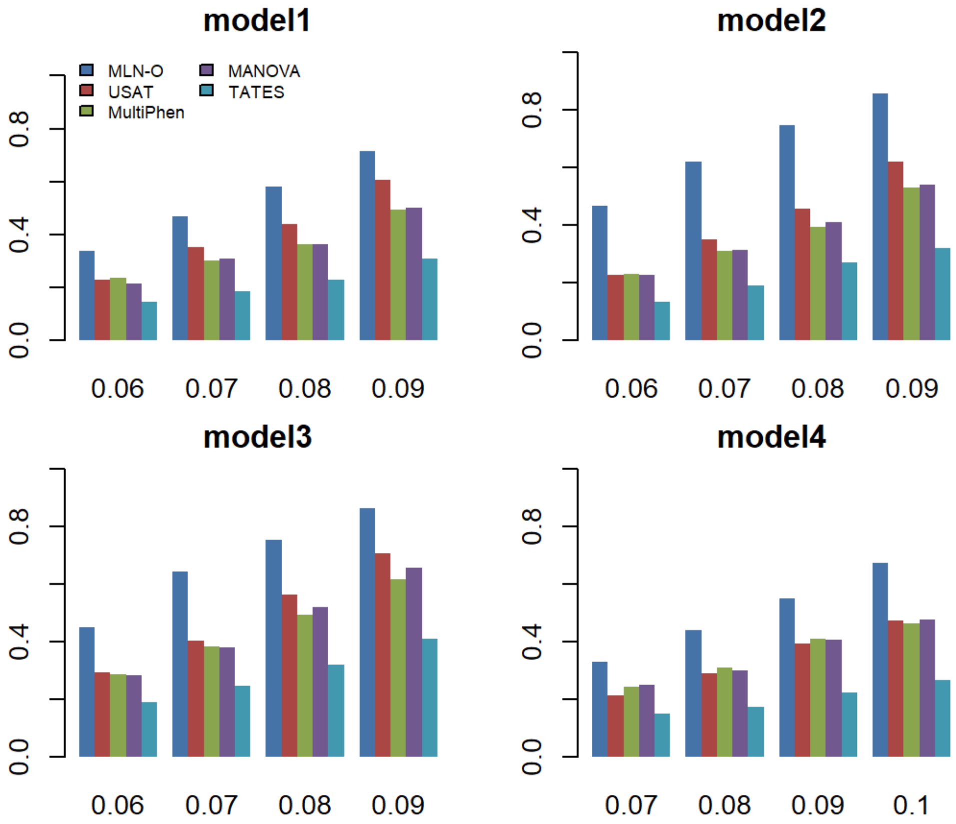

Figure S1. Power comparisons of the five tests (MLN-O, USAT, MultiPhen, MANOVA, and TATES) for 100 binary phenotypes. The sample size is 20,000, case-control ration is 0.001, the between-factor correlation is 0.24, and the within-factor correlation is 0.4.

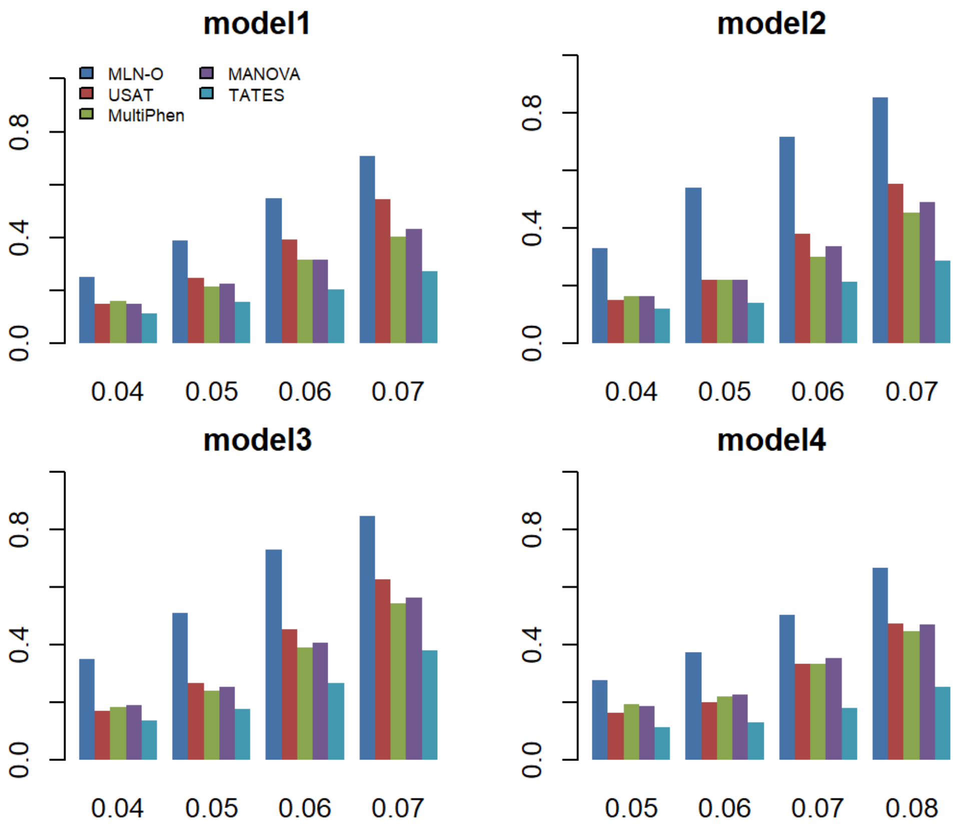

Figure S2. Power comparisons of the five tests (MLN-O, USAT, MultiPhen, MANOVA, TATES) for 100 binary phenotypes. The sample size is 30,000, case-control ration is 0.001, the between-factor correlation is 0.24, and the within-factor correlation is 0.4.

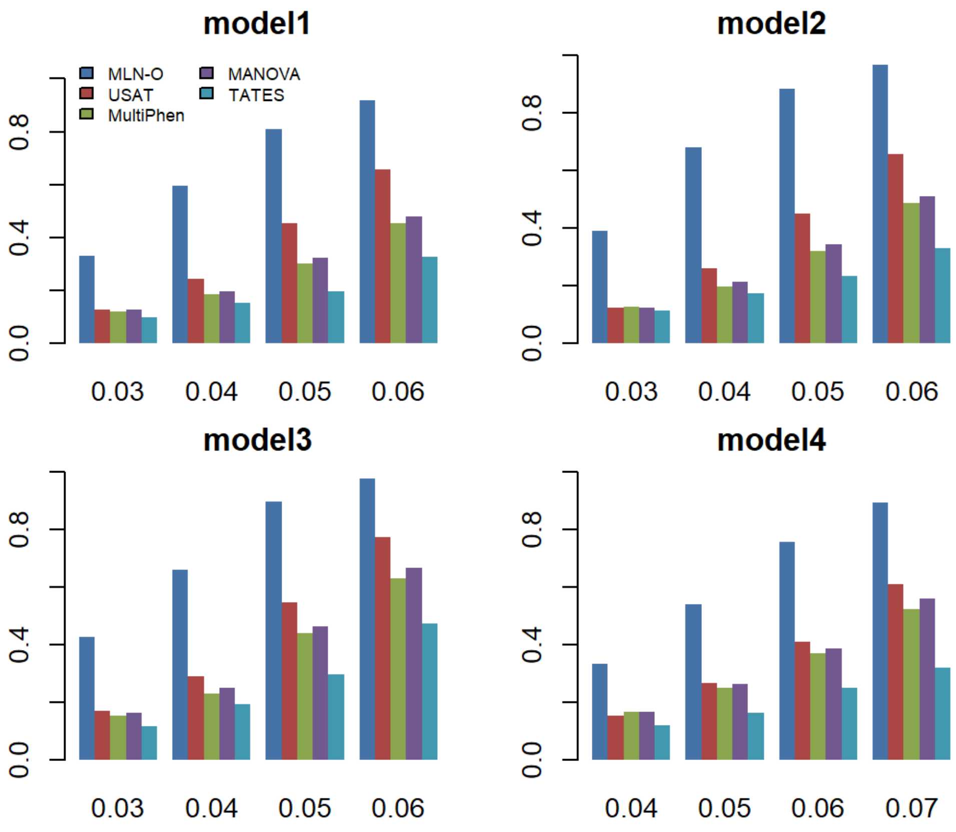

Figure S3. Power comparisons of the five tests (MLN-O, USAT, MultiPhen, MANOVA, TATES) for 100 binary phenotypes. The sample size is 30,000, case-control ration is 0.002, the between-factor correlation is 0.24, and the within-factor correlation is 0.4.

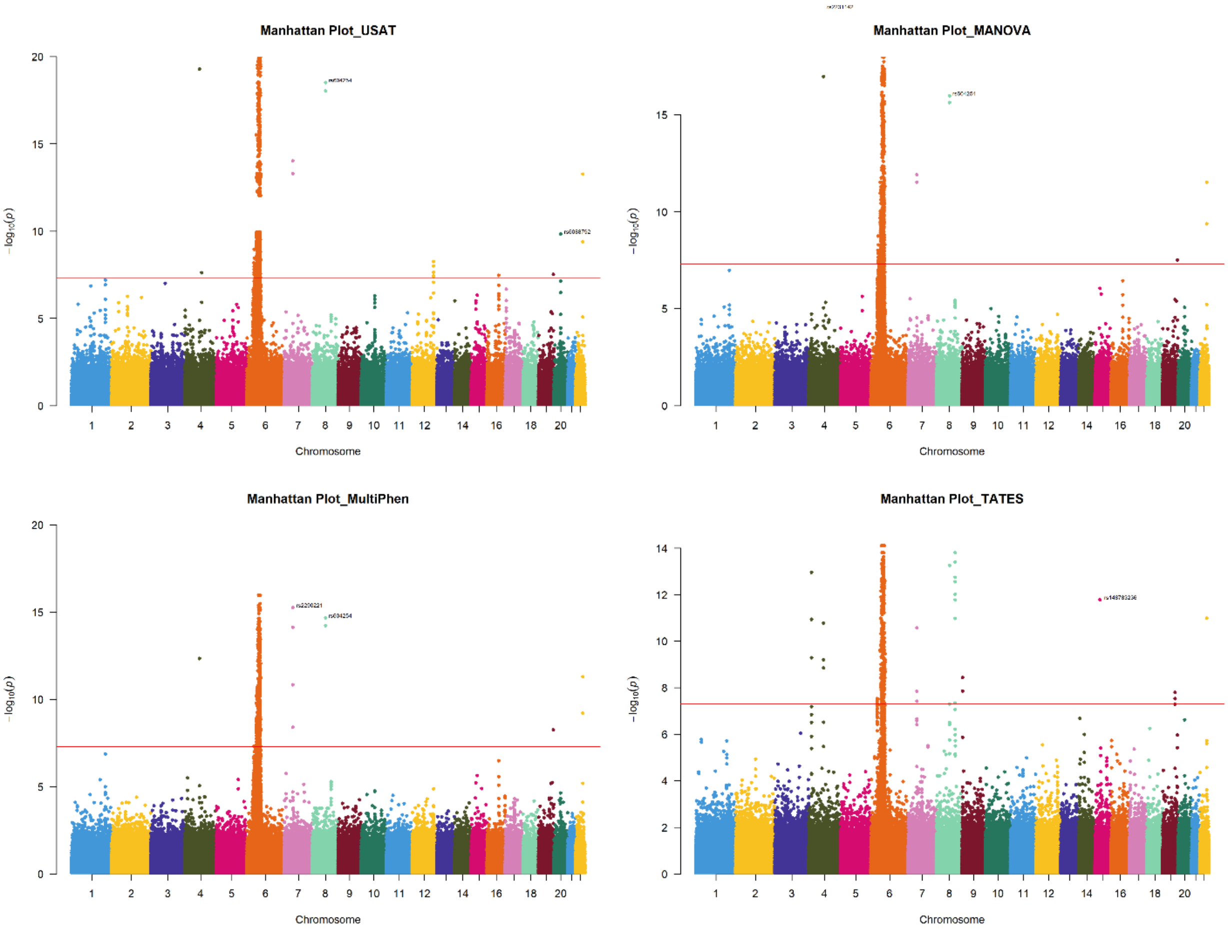

Figure S4. Manhattan plots of negative log-transformed p values of the real data analysis by the four comparison methods (USAT, MANOVA, MultiPhen, TATES) against base pair positions for 22 autosomes . The red horizontal line represents the GWAS significance level.

References

1. Tikkanen, E., et al., *Biological Insights Into Muscular Strength: Genetic Findings in the UK Biobank.* Sci Rep, 2018. **8**(1): p. 6451.
2. Ortiz-Fernández, L., et al., *Identification of susceptibility loci for Takayasu arteritis through a large multi-ancestral genome-wide association study.* Am J Hum Genet, 2021. **108**(1): p. 84-99.
3. Hou, S., et al., *Identification of a susceptibility locus in STAT4 for Behçet's disease in Han Chinese in a genome-wide association study.* Arthritis Rheum, 2012. **64**(12): p. 4104-13.
4. Allanore, Y., et al., *Genome-wide scan identifies TNIP1, PSORS1C1, and RHOB as novel risk loci for systemic sclerosis.* PLoS Genet, 2011. **7**(7): p. e1002091.
5. Renauer, P.A., et al., *Identification of Susceptibility Loci in IL6, RPS9/LILRB3, and an Intergenic Locus on Chromosome 21q22 in Takayasu Arteritis in a Genome-Wide Association Study.* Arthritis Rheumatol, 2015. **67**(5): p. 1361-8.
6. Kunz, M., et al., *Genome-wide association study identifies new susceptibility loci for cutaneous lupus erythematosus.* Exp Dermatol, 2015. **24**(7): p. 510-5.
7. Tangtanatakul, P., et al., *Meta-analysis of genome-wide association study identifies FBN2 as a novel locus associated with systemic lupus erythematosus in Thai population.* Arthritis Res Ther, 2020. **22**(1): p. 185.
8. Yin, X., et al., *Meta-analysis of 208370 East Asians identifies 113 susceptibility loci for systemic lupus erythematosus.* Ann Rheum Dis, 2021. **80**(5): p. 632-640.
9. Hüffmeier, U., et al., *Common variants at TRAF3IP2 are associated with susceptibility to psoriatic arthritis and psoriasis.* Nat Genet, 2010. **42**(11): p. 996-9.
10. Johnson, T.A., et al., *Association of an IGHV3-66 gene variant with Kawasaki disease.* J Hum Genet, 2021. **66**(5): p. 475-489.
11. Styrkarsdottir, U., et al., *Multiple genetic loci for bone mineral density and fractures.* N Engl J Med, 2008. **358**(22): p. 2355-65.
12. Richards, J.B., et al., *Bone mineral density, osteoporosis, and osteoporotic fractures: a genome-wide association study.* Lancet, 2008. **371**(9623): p. 1505-12.
13. Graham, R.R., et al., *Genetic variants near TNFAIP3 on 6q23 are associated with systemic lupus erythematosus.* Nat Genet, 2008. **40**(9): p. 1059-61.
14. Rivadeneira, F., et al., *Twenty bone-mineral-density loci identified by large-scale meta-analysis of genome-wide association studies.* Nat Genet, 2009. **41**(11): p. 1199-206.
15. Han, J.W., et al., *Genome-wide association study in a Chinese Han population identifies nine new susceptibility loci for systemic lupus erythematosus.* Nat Genet, 2009. **41**(11): p. 1234-7.
16. Gateva, V., et al., *A large-scale replication study identifies TNIP1, PRDM1, JAZF1, UHRF1BP1 and IL10 as risk loci for systemic lupus erythematosus.* Nat Genet, 2009. **41**(11): p. 1228-33.
17. Albagha, O.M., et al., *Genome-wide association study identifies variants at CSF1, OPTN and TNFRSF11A as genetic risk factors for Paget's disease of bone.* Nat Genet, 2010. **42**(6): p. 520-4.

18. Hsu, Y.H., et al., *An integration of genome-wide association study and gene expression profiling to prioritize the discovery of novel susceptibility Loci for osteoporosis-related traits.* PLoS Genet, 2010. **6**(6): p. e1000977.
19. Albagha, O.M., et al., *Genome-wide association identifies three new susceptibility loci for Paget's disease of bone.* Nat Genet, 2011. **43**(7): p. 685-9.
20. Estrada, K., et al., *Genome-wide meta-analysis identifies 56 bone mineral density loci and reveals 14 loci associated with risk of fracture.* Nat Genet, 2012. **44**(5): p. 491-501.
21. Yang, W., et al., *Meta-analysis followed by replication identifies loci in or near CDKN1B, TET3, CD80, DRAM1, and ARID5B as associated with systemic lupus erythematosus in Asians.* Am J Hum Genet, 2013. **92**(1): p. 41-51.
22. Paternoster, L., et al., *Genetic determinants of trabecular and cortical volumetric bone mineral densities and bone microstructure.* PLoS Genet, 2013. **9**(2): p. e1003247.
23. Martin, J.E., et al., *A systemic sclerosis and systemic lupus erythematosus pan-meta-GWAS reveals new shared susceptibility loci.* Hum Mol Genet, 2013. **22**(19): p. 4021-9.
24. Cortes, A., et al., *Identification of multiple risk variants for ankylosing spondylitis through high-density genotyping of immune-related loci.* Nat Genet, 2013. **45**(7): p. 730-8.
25. Li, Y., et al., *A genome-wide association study in Han Chinese identifies a susceptibility locus for primary Sjögren's syndrome at 7q11.23.* Nat Genet, 2013. **45**(11): p. 1361-5.
26. Okada, Y., et al., *Genetics of rheumatoid arthritis contributes to biology and drug discovery.* Nature, 2014. **506**(7488): p. 376-81.
27. Kim, K., et al., *High-density genotyping of immune loci in Koreans and Europeans identifies eight new rheumatoid arthritis risk loci.* Ann Rheum Dis, 2015. **74**(3): p. e13.
28. Kemp, J.P., et al., *Phenotypic dissection of bone mineral density reveals skeletal site specificity and facilitates the identification of novel loci in the genetic regulation of bone mass attainment.* PLoS Genet, 2014. **10**(6): p. e1004423.
29. Huffman, J.E., et al., *Modulation of genetic associations with serum urate levels by body-mass-index in humans.* PLoS One, 2015. **10**(3): p. e0119752.
30. Bentham, J., et al., *Genetic association analyses implicate aberrant regulation of innate and adaptive immunity genes in the pathogenesis of systemic lupus erythematosus.* Nat Genet, 2015. **47**(12): p. 1457-1464.
31. Lessard, C.J., et al., *Identification of a Systemic Lupus Erythematosus Risk Locus Spanning ATG16L2, FCHSD2, and P2RY2 in Koreans.* Arthritis Rheumatol, 2016. **68**(5): p. 1197-1209.
32. Pickrell, J.K., et al., *Detection and interpretation of shared genetic influences on 42 human traits.* Nat Genet, 2016. **48**(7): p. 709-17.
33. Pei, Y.F., et al., *Association of 3q13.32 variants with hip trochanter and intertrochanter bone mineral density identified by a genome-wide association study.* Osteoporos Int, 2016. **27**(11): p. 3343-3354.

34. Morris, D.L., et al., *Genome-wide association meta-analysis in Chinese and European individuals identifies ten new loci associated with systemic lupus erythematosus.* Nat Genet, 2016. **48**(8): p. 940-946.
35. Nielson, C.M., et al., *Novel Genetic Variants Associated With Increased Vertebral Volumetric BMD, Reduced Vertebral Fracture Risk, and Increased Expression of SLC1A3 and EPHB2.* J Bone Miner Res, 2016. **31**(12): p. 2085-2097.
36. Taylor, K.E., et al., *Genome-Wide Association Analysis Reveals Genetic Heterogeneity of Sjögren's Syndrome According to Ancestry.* Arthritis Rheumatol, 2017. **69**(6): p. 1294-1305.
37. Terao, C., et al., *Transethnic meta-analysis identifies GSDMA and PRDM1 as susceptibility genes to systemic sclerosis.* Ann Rheum Dis, 2017. **76**(6): p. 1150-1158.
38. Langefeld, C.D., et al., *Transancestral mapping and genetic load in systemic lupus erythematosus.* Nat Commun, 2017. **8**: p. 16021.
39. Medina-Gomez, C., et al., *Bivariate genome-wide association meta-analysis of pediatric musculoskeletal traits reveals pleiotropic effects at the SREBF1/TOM1L2 locus.* Nat Commun, 2017. **8**(1): p. 121.
40. Kemp, J.P., et al., *Identification of 153 new loci associated with heel bone mineral density and functional involvement of GPC6 in osteoporosis.* Nat Genet, 2017. **49**(10): p. 1468-1475.
41. Medina-Gomez, C., et al., *Life-Course Genome-wide Association Study Meta-analysis of Total Body BMD and Assessment of Age-Specific Effects.* Am J Hum Genet, 2018. **102**(1): p. 88-102.
42. Pei, Y.F., et al., *Joint study of two genome-wide association meta-analyses identified 20p12.1 and 20q13.33 for bone mineral density.* Bone, 2018. **110**: p. 378-385.
43. Kim, S.K., *Identification of 613 new loci associated with heel bone mineral density and a polygenic risk score for bone mineral density, osteoporosis and fracture.* PLoS One, 2018. **13**(7): p. e0200785.
44. Laufer, V.A., et al., *Genetic influences on susceptibility to rheumatoid arthritis in African-Americans.* Hum Mol Genet, 2019. **28**(5): p. 858-874.
45. Márquez, A., et al., *Meta-analysis of Immunochip data of four autoimmune diseases reveals novel single-disease and cross-phenotype associations.* Genome Med, 2018. **10**(1): p. 97.
46. Kichaev, G., et al., *Leveraging Polygenic Functional Enrichment to Improve GWAS Power.* Am J Hum Genet, 2019. **104**(1): p. 65-75.
47. Morris, J.A., et al., *An atlas of genetic influences on osteoporosis in humans and mice.* Nat Genet, 2019. **51**(2): p. 258-266.
48. Pei, Y.F., et al., *Joint Association Analysis Identified 18 New Loci for Bone Mineral Density.* J Bone Miner Res, 2019. **34**(6): p. 1086-1094.
49. Hernandez Cordero, A.I., et al., *Genome-wide Associations Reveal Human-Mouse Genetic Convergence and Modifiers of Myogenesis, CPNE1 and STC2.* Am J Hum Genet, 2019. **105**(6): p. 1222-1236.
50. Kwon, Y.C., et al., *Genome-wide association study in a Korean population identifies six novel susceptibility loci for rheumatoid arthritis.* Ann Rheum Dis, 2020. **79**(11): p. 1438-1445.

51. Feng, G.J., et al., *Identification of pleiotropic loci underlying hip bone mineral density and trunk lean mass.* J Hum Genet, 2021. **66**(3): p. 251-260.
52. Wei, X.T., et al., *Pleiotropic genomic variants at 17q21.31 associated with bone mineral density and body fat mass: a bivariate genome-wide association analysis.* Eur J Hum Genet, 2021. **29**(4): p. 553-563.
53. Pei, Y.F., et al., *The genetic architecture of appendicular lean mass characterized by association analysis in the UK Biobank study.* Commun Biol, 2020. **3**(1): p. 608.
54. Ha, E., S.C. Bae, and K. Kim, *Large-scale meta-analysis across East Asian and European populations updated genetic architecture and variant-driven biology of rheumatoid arthritis, identifying 11 novel susceptibility loci.* Ann Rheum Dis, 2021. **80**(5): p. 558-565.
55. Gill, D., et al., *Urate, Blood Pressure, and Cardiovascular Disease: Evidence From Mendelian Randomization and Meta-Analysis of Clinical Trials.* Hypertension, 2021. **77**(2): p. 383-392.
56. Jones, G., et al., *Genome-wide meta-analysis of muscle weakness identifies 15 susceptibility loci in older men and women.* Nat Commun, 2021. **12**(1): p. 654.
57. Zhang, Y.M., et al., *Shared genetic study gives insights into the shared and distinct pathogenic immunity components of IgA nephropathy and SLE.* Mol Genet Genomics, 2021. **296**(4): p. 1017-1026.
58. Sakaue, S., et al., *A cross-population atlas of genetic associations for 220 human phenotypes.* Nat Genet, 2021. **53**(10): p. 1415-1424.
59. Greenbaum, J., et al., *A multiethnic whole genome sequencing study to identify novel loci for bone mineral density.* Hum Mol Genet, 2022. **31**(7): p. 1067-1081.
60. Evans, D.M., et al., *Interaction between ERAP1 and HLA-B27 in ankylosing spondylitis implicates peptide handling in the mechanism for HLA-B27 in disease susceptibility.* Nat Genet, 2011. **43**(8): p. 761-7.
61. Hinks, A., et al., *Dense genotyping of immune-related disease regions identifies 14 new susceptibility loci for juvenile idiopathic arthritis.* Nat Genet, 2013. **45**(6): p. 664-9.
62. Styrkarsdottir, U., et al., *Meta-analysis of Icelandic and UK data sets identifies missense variants in SMO, IL11, COL11A1 and 13 more new loci associated with osteoarthritis.* Nat Genet, 2018. **50**(12): p. 1681-1687.
63. Tachmazidou, I., et al., *Identification of new therapeutic targets for osteoarthritis through genome-wide analyses of UK Biobank data.* Nat Genet, 2019. **51**(2): p. 230-236.
64. Nikopensius, T., et al., *Association analysis of juvenile idiopathic arthritis genetic susceptibility factors in Estonian patients.* Clin Rheumatol, 2021. **40**(10): p. 4157-4165.
65. Boer, C.G., et al., *Deciphering osteoarthritis genetics across 826,690 individuals from 9 populations.* Cell, 2021. **184**(18): p. 4784-4818.e17.
66. Nedoszytko, B., et al., *Results from a Genome-Wide Association Study (GWAS) in Mastocytosis Reveal New Gene Polymorphisms Associated with WHO Subgroups.* Int J Mol Sci, 2020. **21**(15).
67. Astle, W.J., et al., *The Allelic Landscape of Human Blood Cell Trait Variation and Links to Common Complex Disease.* Cell, 2016. **167**(5): p. 1415-1429.e19.

68. Tin, A., et al., *Target genes, variants, tissues and transcriptional pathways influencing human serum urate levels.* Nat Genet, 2019. **51**(10): p. 1459-1474.
69. Aterido, A., et al., *Genetic variation at the glycosaminoglycan metabolism pathway contributes to the risk of psoriatic arthritis but not psoriasis.* Ann Rheum Dis, 2019. **78**(3).
70. Sinnott-Armstrong, N., et al., *Genetics of 35 blood and urine biomarkers in the UK Biobank.* Nat Genet, 2021. **53**(2): p. 185-194.
71. Miller, F.W., et al., *Genome-wide association study identifies HLA 8.1 ancestral haplotype alleles as major genetic risk factors for myositis phenotypes.* Genes Immun, 2015. **16**(7): p. 470-80.
72. Buda, P., et al., *Association Between rs12037447, rs146732504, rs151078858, rs55723436, and rs6094136 Polymorphisms and Kawasaki Disease in the Population of Polish Children.* Front Pediatr, 2021. **9**: p. 624798.
73. Kou, I., et al., *Genome-wide association study identifies 14 previously unreported susceptibility loci for adolescent idiopathic scoliosis in Japanese.* Nat Commun, 2019. **10**(1): p. 3685.
74. Dehghan, A., et al., *Association of three genetic loci with uric acid concentration and risk of gout: a genome-wide association study.* Lancet, 2008. **372**(9654): p. 1953-61.
75. Gutierrez-Achury, J., et al., *Functional implications of disease-specific variants in loci jointly associated with coeliac disease and rheumatoid arthritis.* Hum Mol Genet, 2016. **25**(1): p. 180-90.
76. Nakayama, A., et al., *GWAS of clinically defined gout and subtypes identifies multiple susceptibility loci that include urate transporter genes.* Ann Rheum Dis, 2017. **76**(5): p. 869-877.
77. Nakayama, A., et al., *Subtype-specific gout susceptibility loci and enrichment of selection pressure on ABCG2 and ALDH2 identified by subtype genome-wide meta-analyses of clinically defined gout patients.* Ann Rheum Dis, 2020. **79**(5): p. 657-665.
78. Sandoval-Plata, G., K. Morgan, and A. Abhishek, *Variants in urate transporters, ADH1B, GCKR and MEPE genes associate with transition from asymptomatic hyperuricaemia to gout: results of the first gout versus asymptomatic hyperuricaemia GWAS in Caucasians using data from the UK Biobank.* Ann Rheum Dis, 2021. **80**(9): p. 1220-1226.
79. Hu, H.J., et al., *Common variants at the promoter region of the APOM confer a risk of rheumatoid arthritis.* Exp Mol Med, 2011. **43**(11): p. 613-21.
80. Demirci, F.Y., et al., *Identification of a New Susceptibility Locus for Systemic Lupus Erythematosus on Chromosome 12 in Individuals of European Ancestry.* Arthritis Rheumatol, 2016. **68**(1): p. 174-83.
81. *Genome-wide association study of 14,000 cases of seven common diseases and 3,000 shared controls.* Nature, 2007. **447**(7145): p. 661-78.
82. Plenge, R.M., et al., *TRAF1-C5 as a risk locus for rheumatoid arthritis--a genomewide study.* N Engl J Med, 2007. **357**(12): p. 1199-209.
83. Hom, G., et al., *Association of systemic lupus erythematosus with C8orf13-BLK and ITGAM-ITGAX.* N Engl J Med, 2008. **358**(9): p. 900-9.

84. Chung, S.A., et al., *Differential genetic associations for systemic lupus erythematosus based on anti-dsDNA autoantibody production.* PLoS Genet, 2011. **7**(3): p. e1001323.
85. Gorlova, O., et al., *Identification of novel genetic markers associated with clinical phenotypes of systemic sclerosis through a genome-wide association strategy.* PLoS Genet, 2011. **7**(7): p. e1002178.
86. Eyre, S., et al., *High-density genetic mapping identifies new susceptibility loci for rheumatoid arthritis.* Nat Genet, 2012. **44**(12): p. 1336-40.
87. Saruhan-Direskeneli, G., et al., *Identification of multiple genetic susceptibility loci in Takayasu arteritis.* Am J Hum Genet, 2013. **93**(2): p. 298-305.
88. Lessard, C.J., et al., *Variants at multiple loci implicated in both innate and adaptive immune responses are associated with Sjögren's syndrome.* Nat Genet, 2013. **45**(11): p. 1284-92.
89. Bossini-Castillo, L., et al., *A genome-wide association study of rheumatoid arthritis without antibodies against citrullinated peptides.* Ann Rheum Dis, 2015. **74**(3): p. e15.
90. Govind, N., et al., *Immunochip identifies novel, and replicates known, genetic risk loci for rheumatoid arthritis in black South Africans.* Mol Med, 2014. **20**(1): p. 341-9.
91. Kristjansson, R.P., et al., *A loss-of-function variant in ALOX15 protects against nasal polyps and chronic rhinosinusitis.* Nat Genet, 2019. **51**(2): p. 267-276.
92. Fung, K., et al., *Genome-wide association study identifies loci for arterial stiffness index in 127,121 UK Biobank participants.* Sci Rep, 2019. **9**(1): p. 9143.
93. Traylor, M., et al., *Genetic associations with radiological damage in rheumatoid arthritis: Meta-analysis of seven genome-wide association studies of 2,775 cases.* PLoS One, 2019. **14**(10): p. e0223246.
94. Liu, L., et al., *Twelve New Genomic Loci Associated With Bone Mineral Density.* Front Endocrinol (Lausanne), 2020. **11**: p. 243.
95. Carmona, F.D., et al., *A Genome-wide Association Study Identifies Risk Alleles in Plasminogen and P4HA2 Associated with Giant Cell Arteritis.* Am J Hum Genet, 2017. **100**(1): p. 64-74.
96. Terao, C., et al., *Genetic determinants and an epistasis of LILRA3 and HLA-B*52 in Takayasu arteritis.* Proc Natl Acad Sci U S A, 2018. **115**(51): p. 13045-13050.
97. Li, Z., et al., *Genome-wide association study in Turkish and Iranian populations identify rare familial Mediterranean fever gene (MEFV) polymorphisms associated with ankylosing spondylitis.* PLoS Genet, 2019. **15**(4): p. e1008038.
98. Wiberg, A., et al., *Handedness, language areas and neuropsychiatric diseases: insights from brain imaging and genetics.* Brain, 2019. **142**(10): p. 2938-2947.
99. Cuellar-Partida, G., et al., *Genome-wide association study identifies 48 common genetic variants associated with handedness.* Nat Hum Behav, 2021. **5**(1): p. 59-70.
100. Kim, S.K., et al., *A genome-wide association study for shoulder impingement and rotator cuff disease.* J Shoulder Elbow Surg, 2021. **30**(9): p. 2134-2145.
101. Harley, J.B., et al., *Genome-wide association scan in women with systemic lupus erythematosus identifies susceptibility variants in ITGAM, PXK, KIAA1542 and other loci.* Nat Genet, 2008. **40**(2): p. 204-10.

102. Onouchi, Y., et al., *A genome-wide association study identifies three new risk loci for Kawasaki disease.* Nat Genet, 2012. **44**(5): p. 517-21.
103. Jiang, X., et al., *An Immunochip-based interaction study of contrasting interaction effects with smoking in ACPA-positive versus ACPA-negative rheumatoid arthritis.* Rheumatology (Oxford), 2016. **55**(1): p. 149-55.
104. Julià, A., et al., *Genome-wide association study of rheumatoid arthritis in the Spanish population: KLF12 as a risk locus for rheumatoid arthritis susceptibility.* Arthritis Rheum, 2008. **58**(8): p. 2275-86.
105. Raychaudhuri, S., et al., *Common variants at CD40 and other loci confer risk of rheumatoid arthritis.* Nat Genet, 2008. **40**(10): p. 1216-23.
106. Negi, S., et al., *A genome-wide association study reveals ARL15, a novel non-HLA susceptibility gene for rheumatoid arthritis in North Indians.* Arthritis Rheum, 2013. **65**(12): p. 3026-35.
107. Chung, S.A., et al., *Lupus nephritis susceptibility loci in women with systemic lupus erythematosus.* J Am Soc Nephrol, 2014. **25**(12): p. 2859-70.
